# Is Research on “Synthetic Cells” Moving to the Next Level?

**DOI:** 10.3390/life9010003

**Published:** 2018-12-26

**Authors:** Pasquale Stano

**Affiliations:** Department of Biological and Environmental Sciences and Technologies (DiSTeBA), University of Salento; Ecotekne—S.P. Lecce-Monteroni, I-73100 Lecce, Italy; pasquale.stano@unisalento.it; Tel.: +39-0832-298-709; Fax: +39-0832-298-732

**Keywords:** artificial cells, autopoiesis, cell-free protein synthesis, complexity, liposomes, microfluidics, numerical modeling, origins of life, protocells, synthetic biology, synthetic cells

## Abstract

“Synthetic cells” research focuses on the construction of cell-like models by using solute-filled artificial microcompartments with a biomimetic structure. In recent years this bottom-up synthetic biology area has considerably progressed, and the field is currently experiencing a rapid expansion. Here we summarize some technical and theoretical aspects of synthetic cells based on gene expression and other enzymatic reactions inside liposomes, and comment on the most recent trends. Such a tour will be an occasion for asking whether times are ripe for a sort of qualitative jump toward novel SC prototypes: is research on “synthetic cells” moving to a next level?

## 1. Introduction

The last few years have been characterized by a tremendous increase of interest toward the bottom-up synthetic biology—and in particular toward the construction of cell-like systems [1,2,3,4,5,6,7,8,9,10,11,12,13,14,15,16,17,18,19,20].

**Figure 1:**
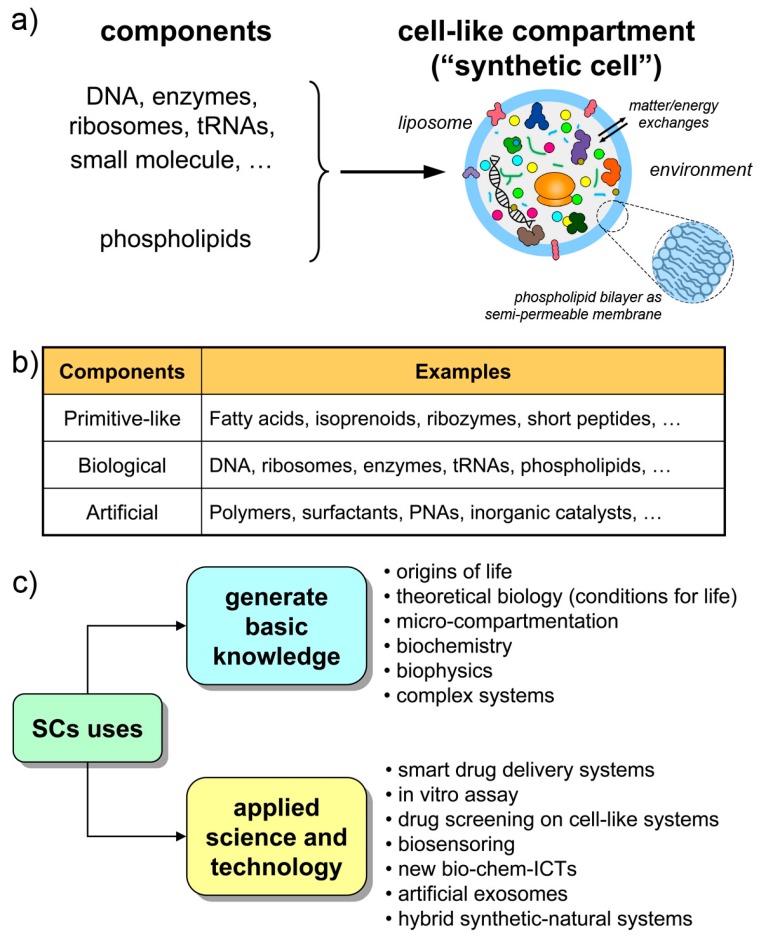
Synthetic cells made by the encapsulation of chemicals inside artificial compartments. (**a**) The case of semi-synthetic cells from biochemical components and liposomes. (**b**) Different types of synthetic cells can be envisaged, depending on the experimental scope. Hybrid systems are also possible. (**c**) Uses of synthetic cells to basic and applied science.

The structures called “synthetic cells”, or “artificial cells”, or “protocells” (sometimes with different nuances in meaning) are chemical or biochemical systems based on micro-compartments that enclose a set of reacting molecules, mimicking the cell structure and behavior. Typically, but not exclusively, lipid vesicles (liposomes) are employed, and biomolecules are entrapped therein depending on the experimental scope (Figure 1a,b). Non-lipidic compartments as well as non-biological components are also used, occasionally mixed in a hybrid design [21,22,23]. Here, we will refer to all these cell-like systems shortly as synthetic cells (SCs), but most of the discussion will be focused on SCs built from biomolecules as DNA, RNA, ribosomes, enzymes, etc. encapsulated within liposomes. The resulting SCs will function by enzyme catalysis and/or by gene expression, closely mimicking biological cells with respect to structure and function. The focus on this approach (the “semi-synthetic” approach [1,6,9]) firstly derives from our involvement in this research, but also considering the great potential of such constructions for basic and applied science.

The Holy Grail of SC research is the construction of *living* SCs [24], a quite challenging goal that must be achieved stepwise (by “living” we mean the capacity of autonomous self-sustainment in an out-of-equilibrium homeostatic state, with the additional possibility of growth-and-division, giving rise to a sort of minimal life cycle, and to evolution). Current SCs have a cell-like structure and perform some life-like operations, but they still resemble biological cells only superficially. The current SC complexity, although is constantly increasing, is quite low. The type of dynamical organization required for self-sustainment by the chemical production of all SC components (the *autopoietic* one, see Section 2.2) is not easy to reach. Contemporary SCs are more similar to heteronomous biochemical reactors (bioreactors), scaled down to the micrometer size, than to actual autonomous living cells. Or, said by a metaphor, they are more machine-like (robot-like) than organism-like [25] (“machine” here is intended in its classical meaning, i.e., a device that is built/programmed to perform certain operations decided by the machine builder).

This scenario is, however, not negative. Current SCs, those within current experimental reach, are very useful in several respects, and help to generate and exploit a vast set of scientific-technological knowledge (Figure 1c). Being structures somewhere located along the non-life/life transition path, current SCs are useful models of primitive compartments, the precursors of primitive living cells. At the same time, their machine-likeness—that is highlighted by terms like programmability, modularity, orthogonality, etc.—makes SCs good tools for understanding-by-building approaches, for biotechnology, or as smart vectors in therapeutic or diagnostic nanomedicine. Finally, current technology is preparatory to more advanced living-like SCs.

At this stage it is important that the field progresses by developing and refining SC technologies and theoretical frameworks. Indeed, SCs research is now a lively and rapidly evolving field. In this paper, we would like to shortly comment on the motivations, the enabling technologies and the current directions of SC research. Needless to say, the text summarizes some of the opinions of the author, and does not claim representing the consensus over the entire (and constantly growing) scientific community. Such a tour, which does not intend to be a comprehensive one, will be an occasion for asking whether times are ripe for a sort of qualitative jump toward novel SC prototypes, which can be emphatically called “SCs 2.0”.

## 2. Why and How SCs? Technologies and Theoretical Frameworks

It could be believed that SCs (of type described in this article) originated in the context of synthetic biology (>2000s), and in particular within the recently emerging bottom-up approaches. Actually, the program of SC construction by assembling separated molecules was clearly defined already in the 1990s, promoted by origins-of-life researchers—mainly the group of Pier Luigi Luisi at the ETH-Zürich [26,27,28,29,30,31,32]. The idea of constructing SCs based on solute encapsulation inside liposomes has its roots in earlier approaches: those promoted by A. I. Oparin should be especially mentioned, based on enzyme-containing coacervates [29,33,34] (a renewed interest toward coacervates has re-emerged recently [35,36,37]). In an origins-of-life context, SCs are intended as models of primitive compartments and primitive cells (protocells) [5,6,7,24,34]. The goal of exploring how cellular life emerged on early Earth from non-living components can indeed benefit from SC approach, because SCs can reveal details about the mechanisms of the formation of early compartments and on the unique features deriving from reaction micro-compartmentalization. It deals with topics like self-assembly of the compartments, solute encapsulation, growth and division, solute partition during division, confinement and crowding effects on internalized reaction, generation and exploitation of trans-membrane gradients, surface-to-volume effects, nutrient permeability and metabolism fuelling, metabolic sustainment, production of internal components, synchronization between solute replication and membrane growth, osmotic forces, role of membrane as matrix or regulator of reactions, and so on: a really rich landscape of peculiarities that had a role (quite probably a determinant one) in the very origin of cellular life. In other words, the SC approach helps unveiling the physico-chemical constraints set by the material nature of a cell on its primeval formation, existence, maintenance and proliferation [38,39,40].

Starting from the early 2000s [2,6,41,42] the original origins-of-life-oriented SC research program partially flowed into synthetic biology, inaugurating, together with other research lines, the so-called “bottom-up” synthetic biology (sometimes also called, again with different nuances in meaning, “cell-free” [43,44], “in vitro” [45,46], or “chemical” [47] synthetic biology). These terms refer, essentially, to a kind of molecular synthetic biology not aiming at engineering (“rewiring”) (micro)organisms, rather at developing from the bottom (from molecules) supra- and multi-molecular systems at varies topological, functional, and hierarchical complexity levels. Bottom-up synthetic biology shares with chemistry (and “systems chemistry” [48]) a constructive attitude.

When it is free from prebiotic questions and constraints, SC research can be expanded in several directions, like, for example, biosensoring or nanomedicine, and can be implemented by using non-biological parts too, as mentioned. Correspondingly, the construction of SCs becomes an interesting target per se, not exclusively intended as intermediate structures on the route toward primitive living cells, or as tool for finding conditions for *minimal* life in a theoretical biology context. In this new inclination, SC research can realize its full potential in new additional scenarios.

### 2.1. Progressing Phases of SCs Research

**Figure 2:**
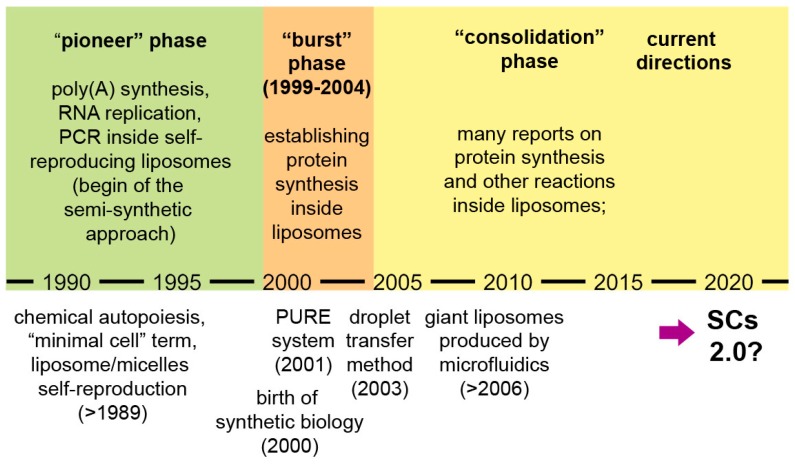
A schematic representation of the evolution of SC research, with a specific focus on chemical and biochemical reactions inside fatty acid vesicles and lipid vesicles (especially, protein synthesis). Emphasis is given to the development of protein synthesis inside liposomes, as a tool for functionalizing SCs. After the consolidation phase, it seems that in the recent years the sophistication of SC systems is rapidly increasing, possibly leading SCs to a next level, “SCs 2.0”.

Broader accounts of research on SCs and related subjects are available [9,18,49]. Here we would like to focus on the progressing phases of compartmentalized reactions and in particular on gene expression inside liposomes. Three periods can be identified (Figure 2):

- a pioneer phase (the 1990s): investigations based on encapsulation of biomolecules inside liposomes, with the explicit intention of creating protocellular models [28,29,30,31,34];
- a “burst” phase (1999–2004): several decisive experimental reports were published, laying the foundations of protein synthesis inside liposomes—a keystone technology for current SCs [3,4,32,50,51,52];
- a “consolidation” phase (after 2004): protein synthesis and other enzymatic reactions inside liposomes and other compartments have been studied in great detail.

The final part of the consolidation phase, which corresponds to the last few years, is a thriving momentum. Quite elaborated SCs have been reported, suggesting that the field is moving toward a qualitative jump that we would like to discuss in this paper. A tremendous increase of interest emerged recently. This attention is not restricted to specific reactions like gene expression, but to several approaches to SCs of any type. This is witnessed by large international projects and initiatives, like the MaxSynBio consortium, the BaSyC project, the CREST-PRESTO funding program by the JST, the “build-a-cell” open science initiative, the Japanese Society for Cell Synthesis Research, the ‘Building a Synthetic Cell’ An Ideas Lab Activity (from NSF), and many others (including past EU-FP6 projects such as PACE and SYNTHCELLS). SC research is definitely legitimized as one of the most important and ambitious synthetic biology projects, being radically innovative with respect to any other pre-existing technology.

### 2.2. Enabling Technologies

The technology for constructing liposome-based SCs operating by gene expression mainly stems from a combination of at least four technological platforms, namely, liposome technology, cell-free systems, microfluidics, numerical modeling.

**Figure 3:**
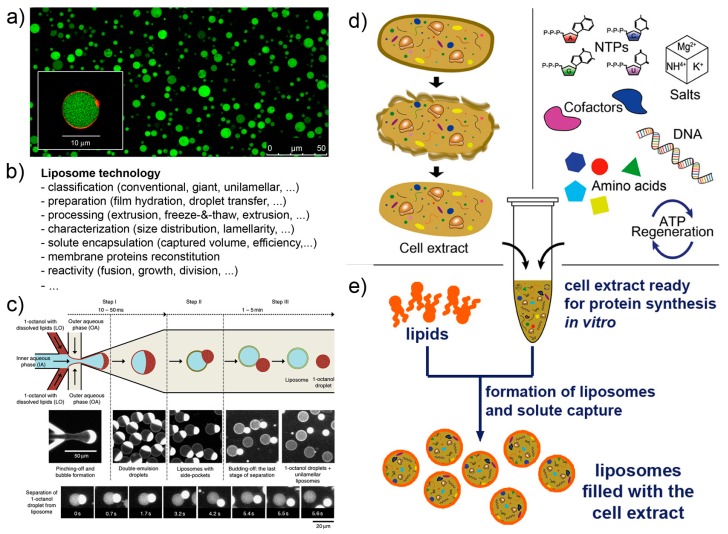
Liposome technology and cell-free systems. (**a**) Giant lipid vesicles are often (but not exclusively) used as compartments for the construction of SCs (image reproduced from [68] published under the CC-BY license). (**b**) A list of issues in liposome technology. (**c**) Giant lipid vesicles (and giant polymer vesicles) can be build in highly controlled manner by modern microfluidic technologies (image reproduced from [69] published under the CC-BY license). (**d**) Cell extracts, typically (but not exclusively) from *E. coli*, are employed as biomolecular systems for performing in vitro protein synthesis. (**e**) When encapsulated inside liposomes (or other compartments) they give rise to cell-like systems (i.e., SCs). Image (**d**) is reproduced, with modifications, from [70] published under the CC-BY license. The reconstituted kit PURE system [71,72], whose composition is known, can be employed in substitution to cell extracts.

Liposome technology (also known as liposomology, Figure 3a,b) is a well-known and robust technology started with the discovery of liposome formation in the 1960s and largely developed in the following decades for studying the biophysical properties of lipids, for reconstituting membrane proteins, and for drug delivery purposes. Several different liposome preparation methods are available [53,54,55,56], as well as procedures for solute encapsulation, post-production processing (sizing, purification, etc.), and measurements (scattering techniques, microscopy, and the recently applied flow cytometry [57,58,59]). Liposome technology allows the formation of conventional (<μm) and giant (>μm [56,60,61]) liposomes in many conditions. The solutes of interest are encapsulated inside liposomes during the very moment of liposome formation, for example when dry lipids are allowed to swell in an aqueous solution. Importantly, a novel method for liposome formation was recently introduced: the droplet transfer method [62,63,64]. It was firstly applied for constructing protein-synthesizing giant liposomes in 2004 [4]. The method deserves a special mention here because it forms giant liposomes in high ionic strength solutions with a very efficient solute entrapment [56,65]. Since then, this method has been largely used; detailed protocols are available [66,67]. Additional knowledge of vesicle growth, pearling, division, fusion, invagination, and responses to osmotic stress enriches the landscape of available tools for SC technology, and it is applied depending on experimental scopes.

Cell-free systems are also known since decades, but the advent of cell-free synthetic biology decisively boosted its further developments, especially in the context of gene expression (Figure 3d,e). Cell-free systems are at the core of SC technology for two reasons. Firstly, they allow “functionalization” of SCs by producing proteins inside liposomes, as it happens for biological cells (e.g., production of enzymes, cytoskeletal elements, membrane proteins); secondly because SCs can host genetic circuits that function as logical gates, whose pattern can be regulated (for example by transcription factors). Transcription factors, in turn, can be also synthesized inside SCs, and being activated by signal molecules. A complex and exciting scenario emerges, which brings about the construction of programmable SCs (and autonomous and autopoietic ones as well [73]). Cell-free gene expression is traditionally based on prokaryote or eukaryote cell extracts. These systems are readily available, but their detailed composition is not easy to determine. In a bottom-up synthetic biology perspective, this lack of knowledge can be seen, in some cases, as a limitation. Cell extracts, on the other hand, perform quite well in terms of protein synthesis, and this feature can overcome the above-mentioned remark. As an alternative to cell extracts, a reconstituted kit, the PURE system, has been introduced in 2001 (based on *E. coli* components) [71,72], and it is commercially available. Although the PURE system is more expensive than cell extracts and performs generally worst (yields of about one third [74]), its major advantage relates to the “*minimal cell*” concept. The PURE system, indeed, has a known and minimal composition in terms of macromolecules required for protein synthesis. The corresponding set of genes required to encode all PURE system components is a subset of the minimal genome [75,76].

The fact that cell-free systems are so relevant for SC technology can be understood by analyzing some of the open issues related to their use. The expression capacity of cell-free systems has been improved [70,77] but the advancements of SC technology require further optimization and better performances. The synthesis of sufficient amounts of proteins in SCs can be indeed a limiting factor in the case of more complex designs, as well as the need of post-translation modifications can be critical in some cases. Recent strategies for facilitating the PURE system production have been reported [78]. These and other advancements will allow more extensive uses of cell-free systems (from scaling-up to high-throughput screening), which in turn can further contribute to the future construction of SCs. 

Microfluidics can be applied to produce SCs [69,79,80,81]. Probably SCs positioning and manipulation will also become relevant in the near future (for example, construction of 2D and 3D tissue-like arrays [82]). Microfluidics integrates liposome technology by providing alternative methods for the production of solute-filled liposomes, in a *high-throughput* and *highly reproducible* manner (Figure 3c). The current strategies for producing liposomes by microfluidics resemble the droplet transfer method. Water-in-oil droplets are easily created in microfluidic channels by injecting an aqueous solution (possibly containing the solutes of interest, i.e., the inner solution) in a continuous flow of an apolar solvent containing lipids (but amphiphilic polymers can be also used [83,84]). The resulting water-in-oil droplets, in turn, are transferred in a continuous flow of an aqueous solution that becomes the outer solution. Water-in-oil-in-water (w/o/w) double emulsion droplets are obtained, whose oil amount greatly varies depending on the type of apparatus, chemicals, operating conditions. The residual oil content in the resulting vesicle-like w/o/w droplets can, in some cases, be very low, and the latter would be very similar to, or essentially indistinguishable from, pure liposomes. Giant vesicles are typically obtained, but a method for producing conventional vesicles has been reported too [85]. The advantages of liposome production by microfluidics lie in the low between-liposomes variability, and in the possibility of entrapping molecules and other particles (however, this can be done also by the droplet transfer method). On the contrary, this technology is not suitable when the goal of an investigation focuses on the investigation of spontaneous mechanism of vesicle formation and solute capture in an unconstrained environment.

Numerical modeling is an important part of any synthetic biology scenario, due to several reasons. First of all, it functions as a guide for experimentalists adopting the design-construction-validation cycle. In a bio-engineering approach, a pre-determined goal can be often reached by this strategy combine experiment and modeling. In SC research, for example, numerical modeling is increasingly used as design tool for genetic circuits [86]. A distinction should be done between in vivo and in vitro systems modeling. In contrary to protein synthesis in vivo, where the gene expression is grafted into a homeostatic metabolic background where regenerative mechanisms for all required components are available, in vitro (cell-free) systems suffer of component weariness, limited resources, and accumulation of reaction products (especially in liposome compartments with a semi-permeable membrane). Accurate modeling cell-free protein synthesis should consider these aspects. Models have been improved thanks to the increasing knowledge of kinetic and thermodynamic constants (for example, see the B10NUMB3R5 initiative) [87,88,89,90,91,92]. Another relevant distinction comes from deterministic vs. stochastic modeling. Although it is true that deterministic rate laws often suffice for simulating the kinetic profile of gene expression, the “digitization” of solutes inside micro-compartments (especially inside sub-micrometer one, like the conventional liposomes) leads to solute partition issues and intrinsic stochastic effects on reaction mechanisms that are quite relevant in SCs studies [49,93,94,95,96]. Numerical modeling is also a valuable tool for simulating molecular communications between SCs or in hybrid SCs-natural cell ensembles [97]. Biophysical modeling can be applied to vesicle transformations, as in the case of SC growth and division [98,99], stochastic solute encapsulation and/or partition [96,100,101,102,103]. Finally, modeling is functional to generate insightful combinatorial experimental designs and analyze high-throughput data.

Although these four technological platforms seems, to date, constituting the core technologies for SCs, this rapidly evolving field could take advantage from other ones, for instance, electronic or photonic interfacing [82,104,105].

### 2.3. Theoretical Frameworks

Researchers interested in building SCs in various scenarios like origins-of-life, biotechnology, molecular communication, and nanomedicine, share the same methodological approach and are also interested, by and large, in similar reactions and similar behavior. There are theoretical frameworks that guide or help the researchers when SCs are especially employed as model of minimal life or primitive life. The “autopoiesis” (self-production) and the “chemoton” model—both from the 1970s—have been considered.

**Figure 4:**
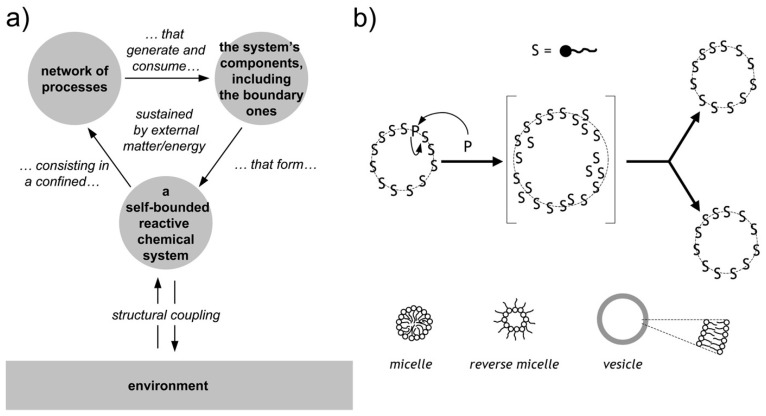
Autopoiesis and minimal chemical autopoietic systems. (**a**) Principles of autopoietic organization. An autopoietic system is defined as a self-bounded chemical system (undergoing continuous transformations) consisting in a confined network of processes that generate and consume the system’s components, including the boundary ones. Note the so-called “structural coupling” with the environment, meaning that the autopoietic system has established (and possibly evolve) its own organization by a dynamical coupling with its environment. (**b**) Minimal autopoietic chemical systems have been generated in the early 1990s employing micelles, reverse micelles, and vesicles (all based on fatty acids). The typical autopoietic dynamics is shown, consisting in growth and division. Image (**b**) reproduced from [110] with the permission of Springer-Nature.

Chemical autopoietic systems are at the roots of the research program that led to SC technology, in the early 1990s. In particular, the first compartmentalized reactions that were investigated with the explicit intention of building cellular models [26,27,28,29,106,107], were inspired by the autopoietic theory of the Cilean biologists Humberto Maturana and Francisco Varela [108,109]. Autopoiesis describes how living cells work from a systemic viewpoint. In particular it focuses on three facts. First, an autopoietic system is a well-defined self-bounded structure in the physical world; it self-determines its distinction/separation from the environment by creating its own boundary. Second, its chemical components—including those of the boundary—are continuously produced and destroyed by a network of processes, at the expenses of energy extracted from the environment (e.g., chemical building blocks). Third, it is capable of maintaining an autopoietic state adaptively in response to perturbations originated in the environment and it is coupled to it. Remarkably, all properties are not determined by any external agent, but are obligate results of the interactions between the components and between the processes of the autopoietic unit, and stem from the autopoietic mechanism itself. Autopoiesis, thus, is a specific type of dynamical organization (Figure 4a).

As mentioned, early work was carried out in the 1990s to build artificial autopoietic systems based on amphiphile-bound cell-like particles, like micelles, reverse micelles, and liposomes (reviewed in [111]), see Figure 4b. Fatty acids were used as amphiphiles, as their structures and properties nicely match (*i*) with the primitiveness plausibility when considered from an origins-of-life perspective, and (*ii*) with the practical requirements of chemical autopoiesis. Fatty acids can be produced by oxidation of fatty alcohol or by hydrolysis of fatty acid anhydrides (or esters). Such simple chemical reactions have been used to generate fatty acids systems that self-reproduce by an autopoietic mechanism.

Does autopoiesis apply to SCs (built by encapsulating biomolecules inside liposomes)? Yes, it does, if the goal is the construction of a system that behaves like a living organism. Starting from the mentioned simple chemical autopoietic systems, the experimental systems were progressively shifted from chemical catalysts to enzymatic ones, inaugurating the research on enzymatic molecular biology reaction inside liposomes [28,29,30,31,32,34]. In this new context, where phospholipids have substituted fatty acids and enzymes/ribosomes/nucleic acids have substituted simple chemical reactants, it is evident that the production of many, or all, SCs components becomes more challenging. This is especially true for liposome-based SCs operating by gene expression. Autopoietic SCs (composed of DNA, RNA, ribosomes, enzymes, lipids, …) require the in situ production of their components. In turn, these production processes require enzymes (and ribosomes) to function, and enzymes (and ribosomes) themselves must be also produced by the SCs, to have a true autopoietic mechanism. This main task requires, in addition, the development of other side-tasks that further complicate the system, forcing an increase of the minimal complexity associated to the primary goal of being autopoietic. A short discussion on this topic is given in Appendix A.

**Figure 5:**
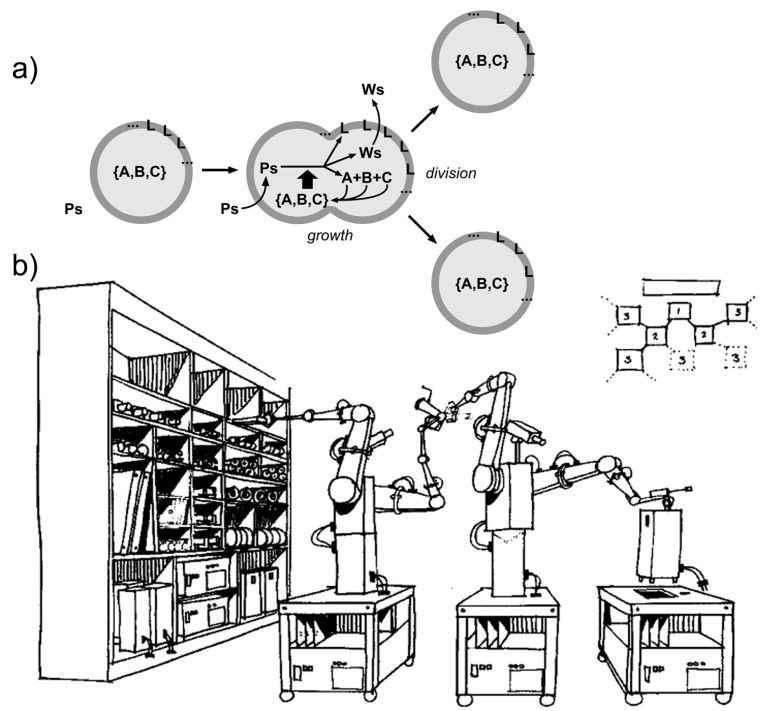
Comparison between (**a**) autopoietic self-reproducing SCs and (**b**) a robotic representation of self-replicating machines, inspired by the von Neumann self-reproducing automata (original title: “Proposed demonstration of simple robot self-replication”, by NASA Conference Publication 2255 (1982), based on the Advanced Automation for Space Missions NASA/ASEE summer study held at the University of Santa Clara in Santa Clara, California, 23 June–29 August 1980). Image (**b**) in the public domain [114]. Note that autopoietic chemical systems produce their components from within.

Note that autopoietic SCs, if they grow and self-reproduce, are conceptually similar to von Neumann self-reproducing automata (Figure 5) (with two remarks: (1) an important practical distinction, i.e., self-reproducing SCs build their own parts internally and therefore produce a copy of itself from within; and (2) a subtle theoretical caveat, i.e., the Turing-computability of autopoietic systems has been debated [112,113], whereas von Neumann automata are Turing-computable by definition).

The chemoton model (fluid chemical automaton) is a chemical model of self-reproducing cells, introduced in the 1970s by the Hungarian biochemist Tibor Gánti [115,116]. The chemoton is designed as three stoichiometrically-coupled autocatalytic subsystems: a metabolic cycle, a template replication system, and a membrane enclosing the other two. The fulcrum of the chemoton is the stoichiometric coupling between the three subsystems, which regulate each other by feedback mechanisms, loops, and stoichiometric cycles (this organization, in essence, is a chemical “clockwork”). The chemoton theory is valuable because it shows that in addition to mechanical and electrical machines, it is possible to design, and in principle create, a fluid (chemical) self-reproducing machine.

Finally, an interesting contribution on the theoretical analysis of the “organisms versus machines” debate in synthetic biology (for example, see the papers in the special issue [117]) has been provided by [25]. According to this analysis, top-down and bottom-up approaches can be depicted as two counter posed strategies. In the top-down approach, organisms are engineered in order to make them more machine-like (programmability, modularity, control, …; in other terms: aiming at constructing “artificial organisms”). In the bottom-up approach, instead, components are first assembled into systems that de facto are essentially intended as machines, with the final goal of rendering these machines more organism-like; in other terms: aiming at constructing “living machines”.

We argue that the current advancements in SC technology will be able to produce complex machine-like biochemical systems. However, to move in the direction of aliveness, relevant aspects of autopoietic theory should be considered. In particular, radical embodiment (all operations are not “computed” by a central processing unit employing representations, but result from the whole SCs multi- and supra-molecular body), enaction (it describes a way of looking at SCs and the “world” in which they exist as a co-evolutive unit that generates SCs perception and cognition), and minimal cognition (intended as the minimal single-cell capacity of co-evolving with its environment by perceiving some environmental changes as perturbations and self-regulating its autopoietic dynamic, to maintain the functional coherence and the coupling) [73,118,119,120,121].

## 3. Current Directions in SCs Research

Many investigations have revealed intriguing physical and chemical aspects of protein synthesis and other reactions inside liposomes. It can be said that the practice of running these types of intra-liposome reactions is generally well understood, at least in its general aspects. The published results in the recent past tell us more, however. It is possible to see the birth of quite interesting trends, all of which can promote a qualitative jump in SC research. “Current directions” includes, for example, the following topics:

- the functionalization of SC membrane
- the *vesosome* architecture
- the community perspective
- the exchange of chemical information

Part of following discussion has been anticipated in a conference paper [122].

### 3.1. Functionalization of SC Membrane

**Figure 6:**
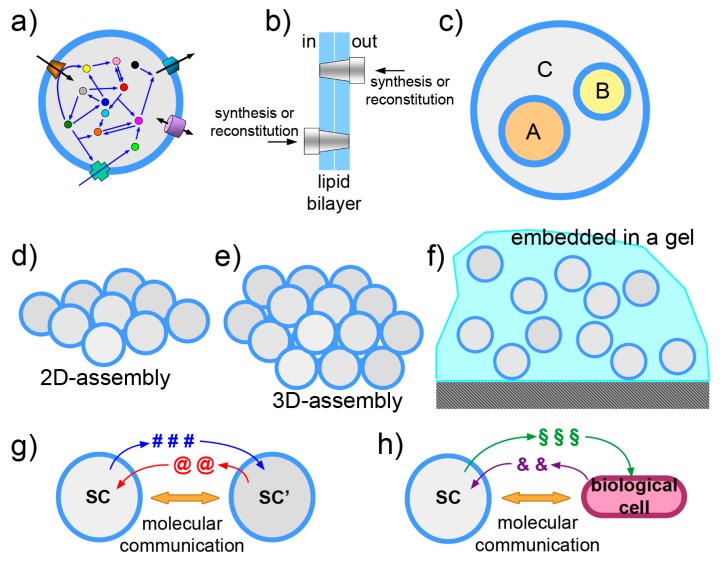
Schematic drawings representing some of the current directions in SC research. (**a**) Functionalization of the SC membrane-by-membrane proteins or similar components. Note that orientation, shown in (**b**), becomes an important issue when dealing with vectorial systems as membrane proteins. (**c**) The nested multicompartment system, or multivesicular vesicle, also known as “vesosome”, is a SC design that allow exploitation of compartmentation, hierarchical levels, chemical gradients across the membranes. (**d**,**e**) Assemblies of SCs in two and three dimensions. (**f**) SCs embedded in biocompatible gel. (**g**,**h**) Molecular communication between SCs or between SCs and biological cells.

Membrane proteins play a major role as receptors, signal- and energy-transducer, transporters. Their huge relevance and the question of their synthesis in SCs has received considerable less attention when compared to water-soluble proteins (Figure 6a). This is due to the intrinsic difficulty of incorporating functional membrane proteins in the membrane. The “reconstitution” of membrane proteins in liposomes—via detergent-mediated processes—is more established [123,124,125], but it may also present technical difficulties. For a pragmatic goal of functionalizing the SCs by membrane proteins, gene expression or reconstitution can be applied, and both from inside or from outside, originating four possible strategies (Figure 6b). However, from an autopoietic perspective, SCs should synthesize their membrane proteins by the internal TX-TL machinery, starting from the corresponding genes.

The mechanism and the requirements of membrane protein insertion in the liposome membrane strongly depend from the protein identity and structure [126]. The latter requires specific protein–lipid interactions and thus determines the insertion, folding, functioning [127]. A first attempt to produce an integral membrane protein by intra-liposome gene expression [128] has taught an important lesson: in order to succeed, three conditions should be simultaneously met: the lipids employed for the SC construction should (a) form good vesicles and entrap the required solutes with high efficiency; (b) not interfere chemically with the protein synthesis machinery; (c) allow the correct insertion and folding of the membrane protein. A number of reports show interesting progresses in membrane protein synthesis—from within: two lipid synthases G3PAT and LPAAT [128], the pheromone receptor and co-receptor BmOR1 and BmOrco [129], the transporter EmrE [130,131,132] membrane anchoring proteins FtsA and ZipA [133] have been obtained in functional form. Detergent-based reconstitution via droplet transfer is another ‘from within’ method, and it has been successfully applied, achieving also high orientations of the protein [134,135] (for other approaches ‘from outside’ see [125,136]).

A key mechanism based on membrane proteins is energy production. This is realized by exploiting a proton gradient, and requires an insulating membrane. ATP synthase, an integral membrane protein, is the key component. SCs functionalized with ATP synthase in the proper orientation can produce ATP in their lumen, in order to fuel other biochemical processes. Clearly, this is one of the next strategic goals. Attempts to reconstitute parts of the ATP synthase machinery have been published [137]. By coupling bacteriorhodopsin and ATP synthase [138] in cytomimetic polymer vesicles, or the photosystem II and ATP synthase [139], it has been shown that it is possible to convert light into chemical energy, i.e., ATP. Systems based on the photosynthetic reaction center, the cytochrome bc1 complex, and ATP synthase are also under investigation [140]. Recent studies have exploited a nested design (small compartments inside large compartments), to build interesting fully-synthetic or hybrid systems that simulate eukaryotic cells [141,142].

### 3.2. The Vesosome Architecture

An intriguing SC design takes inspiration from organellae-containing eukaryotic cells. Liposomes can be constructed in a way that small liposomes (or similar particles) are encapsulated within a larger one (Figure 6c). Technically, these particles are called multi-vesicular vesicles or vesosomes and were originally formulated as drug delivery agents [143,144,145]. They can form spontaneously as unwanted side-products in several vesicle preparation methods, but if they are the main targets, dedicated methods are required. The strategy is to form the outer liposomal shell in the presence of pre-formed small liposomes. This can be done by unrolling calcium cochleate cylinders [146] or by a similar strategy based on interdigitated bilayer sheets [144]. The attractiveness of vesosomes lies in their nested, modular and hierarchical topology. Sub-compartmentation allows control and separation of SC components. If *A*, *B*, *C* are three multimolecular systems each performing a well-defined function (e.g., intended as “modules”), their co-encapsulation inside a liposome leads to a full mixing of components. When a separation of these modules is required, for any reason (chemical incompatibility, cross-talk between the modules, need of a membrane for exploiting chemical gradients, etc.), sub-compartmentation will assure the overall function, provided that relevant chemicals can be exported from the sub-compartments. Examples of this design are known [21,147,148], and the design based on giant lipid vesicles (or giant polymersomes) is currently explored [19,80,149,150,151], also thanks to the availability of microfluidic preparations. In addition to the advantages in terms of design and control, the vesosome architecture is a manner for increasing the overall membrane area per unit of volume. This can be important if major operations have to be played by membrane proteins. In principle, by controlling the fusion between internal vesicles with the outmost membrane, for example with a inducible fusion mechanism [152], a cargo pre-encapsulated in the inner liposomes can be released from the SC in the environment, as it happens in the synaptic cleft. Finally, it should be emphasized that concentration gradients can be exploited thanks to this architecture, as in the case of the above-mentioned ATP synthesis (for example, produced by internalized organellae but in a way that ATP is in the larger compartment).

### 3.3. The Community Perspective

The transition from studying individual, non-interacting SCs to SC “ensembles” is another stimulating frontiers (Figure 6d–f). The SC design can be progressed in order to include also SC communities and between-SC interactions. A SC ensemble can be composed of free moving or immobilized SCs, networked to each other by physical or relational links. Systems of SCs can be imagined in 1, 2 or 3 dimensions, assembled spontaneously, or by microfluidic/micropattern positioning in arrays [153,154,155,156,157,158,159,160,161], or—possibly—by 3D printing. These ensembles can be useful as tissue-like or biofilm-like models and should lead to the emergence of community-level phenomena. Embedding SCs in a gel is also possible (our preliminary observations, partially reported in [67] are quite encouraging: giant vesicles embedded in agar gel matrix are routinely prepared in our lab, displaying excellent stability). With respect to networking, interesting work has been already reported, whereby the nucleic acid functionalization of vesicle membrane allows vesicle linking [162]. Protein synthesis inside such tissue-like vesicle network has been also published [163]. In origin of life research, spontaneously formed SCs “colonies” have been reported [164]. The colonies can be formed by electrostatic interaction between anionic liposomes and poly(lysine), evidencing peculiar properties not present in isolated liposomes. Clearly, a population of objects becomes interesting when there is a kind of physical, chemical or relational interaction among the parts. Synthetic biology allows going one step further, foreseeing interactions that are based on the exchange of information. This consideration brings us straightly to the next topic.

### 3.4. Exchange of Chemical Information

Controlling gene expression by means of transcription factors that respond to chemical signaling is a way for endowing SCs with a sensor/actuator machinery (Figure 6g,h). In this scenario, SCs send and perceive signals to/from the environment, communicate and possibly coordinate their activities. The theoretical and practical implications can be very relevant. A technology based on molecular communication firstly adds to basic communication and information theories [165,166], providing a new paradigm with specific strengths (and limitations). Second, from the applicative side, such communicating SCs can be designed for nanomedicine purposes. Chemical communication is a manner—the most obvious, perhaps—to interface SCs with biological cells. The more general subject of bio-chem-ICTs involves biological, chemical, electronic and hybrid approaches [167]. International projects have already focused on this emerging area (cobra-project.eu, fet-circle.eu).

In 2012 we explicitly defined a research program based on SCs/biological cell interfacing [168] (Figure 6h), inspired by previous seminal work [169,170,171,172]. Since then, several experimental papers have been published, most of which are based on quorum sensing signaling. Such a topic seems very promising, as remarked in a recent review [173]. For example, SCs have been built in order to “translate” a chemical signal for *E. coli* [174], to send signals to *P. aeruginosa* [67], or to establish bi-directional communication with *V. fischeri* [175]. Between-SCs communication has been reported as well [17,176,177].

**Figure 7:**
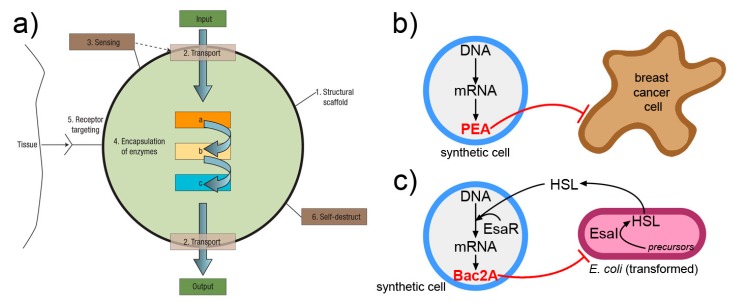
Interesting approaches for interfacing and exploiting SCs operation in a biological and nanomedicine context. (**a**) The “nanofactory” proposed by LeDuc and collaborators [170] can recognize a tissue, sense its environment, activate internalized enzymes, and produce a compound of biomedical utility (image reproduced by [170] with the permission of Springer Nature). (**b**) SC that produces, by gene expression, the *Pseudomonas* exotoxin A (PEA) and kills breast cancer cells in vivo [178]. (**c**) SC that senses homoserine lactone (HSL) signals from *E. coli* and consequently activates its own gene network that ultimately produces a toxin Bac2A, killing the bacterium [179].

A nanomedicine scenario can be derived from the one lucidly presented by LeDuc in 2007 [170], and would involve SCs that, upon receiving an activation message, do diagnostic or therapeutic operations (Figure 7). Two interesting reports should be mentioned, because they have demonstrated how SCs can produce a cytotoxic protein (exotoxin A) in vivo, when injected into the tumor [178], and SCs that, by replying to a signal of bacterial origin, produce the Bac2A antimicrobial peptide that actually kills the bacterium [179]. It is possible that more work in this direction will lead to next-generation smart drug delivery systems based on SC technology.

Parts of the scientific community of network- and communication-engineers have been involved in a foundational activity devoted to expand the classical theory of information and communication to the realm of molecular communications [165]. The rather technical treatment aims at defining the chemical equivalents of concepts and metrics of electromagnetic signals. The challenges come from the several inescapable differences that exist between electromagnetic and chemical signals, with respect to signal, type of information, type of propagation (diffusion), propagation speed/range, propagation medium, energy requirements, elements and mechanisms of the transmission channel (sensor, controller, actuators, etc.). In particular in the world of molecular communication among “nanomachines” (the SCs, or their parts) a critical aspect derives from molecular discreteness, and from the stochastic nature of random processes at the molecular level. Mastering and programming molecular communication, from the bio-chemical and engineering viewpoints will provide a powerful tool for applications based on SC coordinate behavior.

## 4. A Qualitative Jump toward SCs 2.0?

The research on SCs and on similar themes is generating a movement and an attention that were difficult to imagine up to few years ago. To date, several individual reactions, or “modules” composed by several reactions, have been carried out inside lipid vesicles. It is not the aim of this paper to provide a full list of these advancements. An extensive review is available [9], and several newer reports include the systems described in Section 3.1, reactions like PCR [31,180], RT-PCR [181], DNA replication [182], lipid synthesis [128,183], and several others. Each of the reactions can be seen as a kind of “gear” for developing more complex functions in SCs. These studies were carried out according to diverse motivations, but share a common technological framework.

It is expected that we will be able, soon, to prototype increasingly complex SCs. Will we have, then, SCs 2.0? In what sense, and to what extent, does the “2.0” label apply to SCs?

To answer these questions, inevitably, two problems arise. The first is the definition (and possibly the quantification) of SC complexity, the second is the identification of a complexity threshold above which SCs are intended as “2.0”. Both are difficult-to-reply open questions, but whereas the second just requires a consensus within the community of SC researchers, the first one is more fundamental, because defining and quantifying the complexity of any system is notoriously a quite difficult task [184,185]. A plethora of approaches are known, each of them being best applicable to specific problems [186]. The following discussion contains some inputs, and more comments are given in Appendix B. At this preliminary stage the goal is not to provide answers, but to highlight the topic in order to stimulate further investigations.

It seems natural to focus on two specific features of SC, such as *structure* and *organization*.

### 4.1. Structure

From a purely structural viewpoint, the many SCs constructed so far are generally simple. Most of their complexity typically lies in the number and structures of the reacting molecules (e.g., think to protein synthesis), but the SC structure can be typically described just as an isolated and generally inert lipid micro-bag filled with solutes. The droplet transfer method and the microfluidic devices have improved of the efficiency of solute encapsulation (and microfluidics has also reduced the between-SCs variability). However these technical advancements, per se, do not bring SCs at a 2.0 level because the SCs produced by these two methods are not qualitatively different that those produced by other methods (e.g., the classical film hydration method).

On the contrary, the recent trends involving nested vesosome design and SC ensembles go in an interesting direction because they create hierarchical levels, such as:

1. SC ensemble/community
2. individual SCs
3. intra-SC compartments

The resulting systems are without a doubt at a higher complexity level (see Appendix B). These emerging approaches allow the establishment of more complex dynamics and organization, and open to modularity and community behavior. Intriguingly, even when ensembles of non-interacting SCs are considered, population-level phenomena can still emerge, for example the competition for resources [187,188,189], caused by SC diversity [49,95,101,190,191,192]. Physically interacting SCs, as in the above-mentioned example of giant vesicle “colonies”, display interesting properties derived from the mechanism of attachment [164].

SC functionalization with membrane proteins is another milestone on the route to SC 2.0. This is a challenging step, but also a rewarding one, as evidenced in Section 3.1. It requires, in turn, a more accurate management of membrane protein insertion (and the issue of orientation, see Figure 6b), the capacity of constructing SCs with membranes composed of mixed lipids, and asymmetric membranes [64]. The SCs makers have not yet faced these advanced issues in a systematic manner.

### 4.2. Organization

It would be a mistake, however, thinking about the complexification of SC structure without an accompanying turn in their dynamical organization. By organization we mean the network of functional and causal interactions that gives rise to a dynamical pattern (for example, response to stimuli, growth, division, logical operations, etc.). Membrane proteins, internalized organellae-like compartments, SCs ensembles and between-SC interactions are needed for constructing SCs that can operate in more complex manner. This implies a change in the SC organization, changing the number and the type of causally-related processes.

For discussing a qualitative jump of SC organization, one should ask whether or not the internalized components, the subsystems (modules), the liposome shell, and the environment (including other SCs), interact with each other, displaying any kind of system-level features. The idea is that the 2.0 label applies to SCs whose dynamics results from an increasing number of causal relationships between the parts or between the functions of the parts, at the same (or different) hierarchical level(s). Such interactions can lead to feedback, control, cooperation, competition, evolutionary mechanisms, just to mention some. The design and the realization of SC 2.0 should surpass biochemically complex, yet organizationally simple, dynamics. The qualitative jump can be depicted considering either physical interactions, either logical operations occurring in SCs.

To evaluate the complexity of SC organization various approaches can be recalled, for example by converting the reactions occurring in the SC in a network, or translating what SCs do into algorithms. In both cases, several metrics are available to measure the graph complexity or the algorithm complexity [186] (for specifications and caveats see Appendix B).

For example, gene expression under control of T7 promoter and T7 RNA polymerase is a simple and common design. Protein synthesis is activated just by raising the bath temperature from ice-chilled to room temperature or above. In this case the SCs works essentially as a two-states switch (1 bit of information: 0 = off, 1 = on). The performance of such kind of SCs can be rendered by a IF-THEN-ELSE instruction (IF the bath temperature is above a certain value, THEN start the gene expression, ELSE stand-by).

Many of the early works on protein synthesis (or other enzymatic reactions) inside SCs correspond to systems of this (low) complexity, because the goal was just proving that those reactions occur in certain particular conditions. There have been, however, several examples where SCs operate in more complicated way, via multi-step mechanisms that involve the physical interaction of different components, processing chemical signals, performing logical operations.

**Figure 8:**
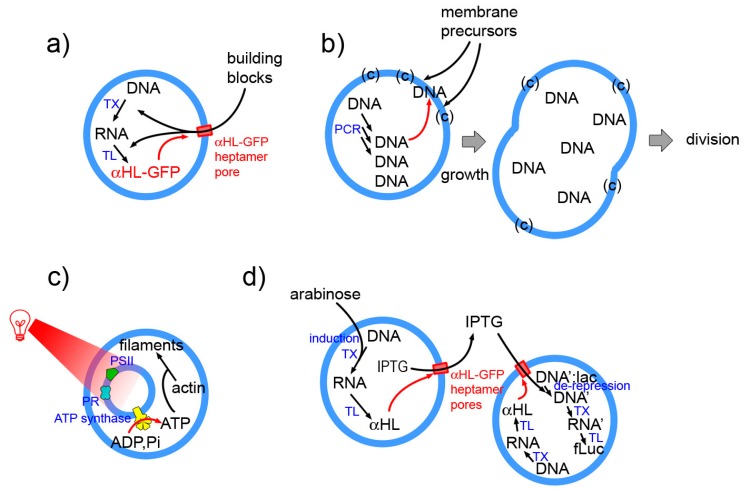
Four selected examples of SCs whose behavior is systemic. (**a**) The α-HL-GFP chimeric protein is produced by TX-TL reactions. It inserts spontaneously in the liposome membrane, forming a pore. Externally present building blocks (and small internal by-products) can pass through the pore (cut-off 3 kDa) allowing a prolonged protein synthesis [4]. (**b**) DNA is produce inside SCs formed by an ad hoc designed membrane (mixture of several components). Anionic DNA interacts with the cationic membrane and catalysts and facilitates the further binding of membrane precursors, ultimately leading to SC division [14]. (**c**) Artificial photosynthetic small compartments, including photoactive proteins and ATP synthase have been encapsulated inside a large compartment. Following actinic irradiation with red light, ATP is produced from ADP and inorganic phosphate. The SC uses ATP for polymerising actin into filaments [142]. (**d**) Upon arabinose activation, SCs of first type produce α-HL, so to form a pore in the membrane. IPTG, which was contained inside these SCs could escape reach a second SC (of different type), which constitutively produces α-HL. Therein, IPTG activates gene expression leading to a final bioluminescence response [17].

Examples as those indicated in Figure 8 (details in the caption) epitomize the essence of next-level SCs. These will be structures with higher organizational complexity, whereby couplings are established between sub-systems at the same or different hierarchical levels, or with the environment. For example in the Noireaux and Libchaber 2004 paper [4] protein synthesis leads to pore opening in the membrane, allowing building blocks entering the SC. The work from the Sugawara group is based on the locally cooperative interactions established by internally synthesized DNA, membrane precursors and lipid catalyst, and leads to SC division as a *systemic* response [14]. The multicompartment (vesosome) design is instead exploited by Lee et al. [142] who “energized” their SCs by internal production of ATP. Finally, an elaborated SC system composed by two SC populations, capable of exchanging chemical information has been reported by Boyden and collaborators [17].

Accordingly, it can be concluded that the transition toward SCs 2.0 is already started, because the current degree of SCs sophistication certainly include several examples of SCs exhibiting system-level patterns. Times are ripe for designing SCs displaying features as the above-indicated ones (feedback, control, cooperativity, communication, organization, etc.). Long-term goals such as full self-maintenance, self-production of the components (autopoiesis), or self-reproduction of the whole SC structure are instead very challenging, and require extensive networking between processes. Realistic goals for the next future refer to intermediary systems of moderate complexity, so to proceed stepwise.

Finally, note that looking at SC organization from a system-level perspective is an essential trait of the *sciences of the artificial systems*, which include hardware, software and wetware approaches (respectively, robotics, artificial intelligence & artificial life, and synthetic biology [73,193]).

## 5. Concluding Remarks

In this paper we have shortly recalled the major techniques and the current research directions in SC research—with an explicit focus to SCs based on gene expression.

The semi-synthetic approach uses powerful molecules (enzymes, ribosomes, RNA, DNA, …) operating with high specificity, which render the construction of SCs an exciting research arena, rich of potentiality in basic and applied science.

SC technology will benefit of further advancements in the four major technologies mentioned in Section 2.2 and will explore many new directions, as those indicated Section 3, and many others as well. However, a key ingredient for the diffusion of SC technology is *reproducibility*. Lack of reproducibility is a limitation that can occur in young technologies. The standardization of parts and protocols, as well as “open science” initiatives will enormously contribute to the between-laboratory reproducibility, bringing about the growth and to the success of SC research. This is an essential condition for broadening and consolidating the community of scientists working of this fascinating topic.

Is the field mature enough to face a qualitative transition, toward, let us say, “SC2.0”? Intuitively, SC complexity is related to system-level behavior, originated from a dynamical organization among SC components, structures, and processes. Experimental records suggest that the transition toward ever more functional and complex SCs is already occurring, although the definition and quantification of SC complexity is a still unexplored topic (this paper, then, calls for future in-depth theoretical analyses on this subject).

More generally, here we have highlighted a remarkable momentum from which, it is hoped, insightful knowledge and outstanding technologies will derive.

## Appendix A. Autopoietic SCs Based on Gene Expression?

The production of proteins inside SCs can be achieved by employing the “PURE system” [194,195], a reconstituted kit for TX-TL reactions with a known and *minimal* composition:

- 36 proteins (20 amino acyl-tRNA synthetase, 10 translation factors, 4 energy-related enzymes, T7 RNA polymerase, methionine trans-formilase)
- *E. coli* ribosomes (composed by 3 rRNAs and 55 ribosomal proteins)
- tRNA mix from *E. coli* (46 according to [196]);
- small molecular weight (MW) compounds (amino acids, nucleotides, etc).

When compared to cell extracts, the PURE system leads to lower yields (as mentioned, ca. one third [74]), but its major advantage refers to a *Gedankenexperiment* with relevance for the construction of autopoietic or quasi-autopoietic SCs.

It is possible to imagine SCs that contain the PURE system and an artificial genome encoding for all macromolecular components of the PURE systems. This genome would be constituted by about 140 genes: a figure that is about 70% of the minimal genome, estimated as 204 genes [76]). Note that the construction of such an artificial genome is not unrealistic after the well-known experiments of the Craig Venter team in 2010–2016 [197,198].

Next, under the very permissive hypothesis that gene expression regulation and post-transcription/post-translation modifications are not strictly required, the result of the PURE system TX-TL reactions would be the entire set of components of the PURE system itself, provided that enough small MW components are available and by-products removed (not to poison the reactions). The resulting hypothetical SCs would still require macromolecular components to replicate DNA, and to produce lipids, but—at least in principle—should be able to produce all PURE system components. This minimalistic scenario should give, then a quasi- autopoietic SC where all internal components, but DNA and lipids, are self-produced. The entire mechanism would be made possible by a continuous supply of small molecules from the environment, and a release of waste chemicals, for example by small pore in the membrane (e.g., the α-haemolysin pore). Such a pore, however, would not allow the release of important information-carrying and function-carrying macromolecules in the environment [4].

One of the several potential hurdles is the synthesis and assembly of functional ribosomes in situ, but encouraging results have been reported recently [199,200,201]. The continuous production of internal components, without compensative degradation pathways (as requested by autopoiesis) excludes the possibility of a homeostatic state, favouring a growth phase. However, the resulting system could not undergo growth-and-division, because lipids are not synthesized. There are experimental indications that the machinery for lipid synthesis can be functional in vitro [28,128,183,202,203], and thus such a “module” can be added to the hypothetical system shown above. In proper conditions, which till now have been demonstrated only for fatty acid vesicles [107,204,205,206,207,208,209], and not for pure phospholipid vesicles, the spontaneous division of growing SCs could be obtained. Finally, DNA replication would be required. As mentioned, a recent report also indicates that it is possible to rely on machinery synthesized in situ for DNA replication (ca. 20 kbp) [182].

The participation of the environment as a source of small MW nutrients and as a sink for byproducts recalls an important consideration, which in the autopoietic theory plays an important part. Living systems, as we know them, live in a specific environment. Their internal mechanisms not only depend from the available “building blocks” found in the environment (and from the energy extracted from the environment), but are adaptively and evolutively *coupled* with environment composition, transformations, and fluctuations (in autopoietic terms: engaged in a “structural coupling”). The organism/environment duality, indeed, is just a result of our operation of “distinction”, which literally creates in our understanding—the observers’ understanding—the organism and the environment as two entities. However, they are not. The functional coupling of an organism with its environment and environmental fluctuations is inseparable, and is at the basis of biological—i.e., autopoietic—cognition. Such an additional aspect becomes fundamental when aiming at constructing artificial *living* systems [73,119,120].

## Appendix B. SC Complexity: A Still Unexplored Topic

Defining and measuring complexity in physical, chemical, and biological systems is a challenging task. As mentioned, there is no universal answer, but different definitions and measurements are best suitable for certain systems rather than to others [184,185,186].

On the other hand, the exercise of focusing on SC complexity, even if not leading to a definitive answer, can be useful to better defining, considering, evaluating what is behind the enterprise of constructing SCs, exploring additional facets that can inspire further investigations.

Here we would like to briefly comment on a couple of possible approaches of SCs complexity, based on structure and organization. The approaches will involve operational strategies where informatics and computer science can be of great help. (*Note*: the discussion has only pragmatic scopes: it is not intended as a contribution to the long-standing debate on the cell/computer or brain/computer analogies [210,211,212,213,214]).

*Structure*. Information is needed to describe the SC structure, at different levels. For example, at the molecular level, to describe DNA genes, RNAs, enzymes, small MW molecules, information refers to the number and the types of components, and to their molecular structure (e.g., size, sequence, connectivity between atoms). At the level of reaction networks, the conversion of one chemical species into another can be seen as a set of sets of vertices connected by edges—a graph, whose complexity can be also determined. Here we will deal with the compositional/topological information associated to the SC level. The SC structure can be described operatively as the set of instruction needed to build a static model of it (e.g., by a computer). The *amount of information* associated to the construction of the SC, and therefore the complexity associated to it, can be discussed in an analogous way to a proposed method for estimating the phenotypic complexity of organisms [215]. In particular, the complexity of an object can be measured by the amount of information required to offset the Shannon entropy of the randomly constructed equivalent objects (i.e., those that could have been originated instead of the target one). Accordingly, the information value (in bits) results to be log_2_ *N*, where *N* is the number of different, but equivalent, randomly constructed objects (giving the same probability to all possible structure is equivalent to say that no previous knowledge is available; details can be found in [215]). Some examples can clarify this approach.

Consider a target SC consisting of one water-soluble molecule A enclosed inside a liposome. The construction of the SC is conceptually made in two steps. A compartment is firstly created, originating a division of the e space in an inner and an outer space. Next, A is located inside the compartment. This corresponds to a dichotomic decision, but a random choice could have lead also to A located outside the compartment. There are *N* = 2 equivalent randomly generated objects that can be constructed, and thus the amount of information required to describe the building of the target SC is 1 bit. Similarly, the amount of information associated to a SC enclosing a water-soluble molecule A and a membrane-embedded molecule B that can stay in two possible orientations, inward and outward, is 2 bits (*N* = 2^2^). If there are *n* molecules A and *m* molecules B, the number of randomly constructed SC configurations becomes 2*^n^* · 2*^m^*, and the amount of information correspondingly increases to *n* + *m*. Due to the its logarithmic nature, information is additive and the several contributions can be calculated separately as far as they refer to independent events.

As a second example, consider a multi-compartment SC (a vesosome) consisting of a large vesicle *V* containing an inner vesicle *v*. Again, 1 bit is required to describe the resulting structure. Note that the space is now divided in three sub-spaces (*v*, *V-v*, and the external space). Suppose that the target SC requires two molecules, A, and B to function, and that a specific configuration is needed. For example, A should be inside *v*, and B inside *V-v*. To compute the amount of information needed to describe/build the target SC the total number of possible combinations is needed. In this case *N* = 2 · 3^2^ = 18 and the amount of information (log_2_ 18) is 4.17 bits (5 bits).

*Organization*. Following a similar operational approach, the complexity of SC organization, intended as its dynamical pattern, can be estimated from the complexity of the network of chemical reactions that occur in the SC, or from the complexity of the algorithm that describes how the SC operates from a logical viewpoint. In both cases, the network graph, or the algorithm should be able to generate a computer model of the physical SC. The literature about network complexity is huge, and there are several metrics available [186]. Similarly, there are several measures of the algorithm complexity. When adapted to SCs, the length of the shortest program that produces the behavior of the target SC can be recognized as a definition analogous to the Kolmogorov–Solomonoff–Chaitin complexity, introduced in the 1960s for strings of symbols [216]. Cyclomatic complexity is another metrics, based on the number of linearly independent paths in an algorithm [217].

Whereas the network approach appears quite general, an important caveat about the algorithm approach refers to the implicit hypothesis that SCs work as “information processing machines” with a finite number of states/pathways. This is actually the case of the currently studied SCs (e.g., logical genetic circuits implanted inside SCs), whose functioning can be indeed simulated by an algorithm: their behavior is Turing computable. On the other hand, because the Turing computability of truly autopoietic systems has been questioned [112,113], the algorithmic complexity should be cautiously considered.

Section 4.2 reports, as an example, the case of SCs that produce proteins by a gene expression mechanism based on the T7 promoter and thermal activation (at low temperature, gene expression is essentially latent). When looked from the network viewpoint, the complexity of gene expression network is obvious, involving about hundred chemicals as starting chemical species, and several hundreds intermediates. However, when the information processing operation of the SC is considered, the system essentially responds to a temperature increase by activating (a very complicated) reaction. In this case, the algorithm that generates the SC pattern is a simple IF-THEN-ELSE instruction. This type of SC operates by just taking one decision, and the space of the possible different patterns includes just two alternative routes (its cyclomatic complexity is 2). SCs that rely on more complex genetic circuits will correspondingly have higher algorithmic complexity; for example, SCs enclosing genetic circuits functioning as an AND gate, producing a chemical species only if two “activators” are simultaneously present. An example can be the synthetic AND genetic gate reported by Noireaux [218], which produces a reporter protein only when the *σ*^54^ transcription factor and the NtrC regulatory proteins are present. For SCs operating in this, or in similar, manner, algorithmic complexity can be used to rank SCs by their complexity, because the comparison is made within the same given frame of description.

However, some interesting investigations have shown SCs, whose behavior differs from a synthetic step that is an end in itself. Four of them have been visually represented in Figure 8 (a short description is given in the figure caption). The common trait of these SCs, that we intuitively see as more complex, is that their behavior is not realized by a mere complexification of the internal genetic circuit, but thanks to *interactions* between different SC parts (or between the SC and the environment), or by a *hierarchical* organization. Hierarchy and interactions are at the core of any organized system, and are the signatures of complexity; they give rise, ultimately, to emergent properties. A network analysis is probably able to identify these features of SC organization. It is then left as open question the problem of defining what is complexity in the SC context and how to measure it, which strategy is best suitable and in which context. As mentioned, this proposal is mainly intended as a stimulus for further investigations on the subject.

A final remark should be done about nomenclature. Here, the word “complexity” has been used according to its loosely defined meaning. Probably this is acceptable owing to the fact that the discussion is still at a very preliminary level. Actually, a distinction should be done, narrowly speaking, between a complicated system and a complex system, especially when dealing with definitions and quantifications.

## References

[1] Luisi P.L. (2002). Toward the engineering of minimal living cells. Anat. Rec..

[2] Pohorille A., Deamer D. (2002). Artificial cells: Prospects for biotechnology. Trends Biotechnol..

[3] Nomura S., Tsumoto K., Hamada T., Akiyoshi K., Nakatani Y., Yoshikawa K. (2003). Gene expression within cell-sized lipid vesicles. ChemBioChem.

[4] Noireaux V., Libchaber A. (2004). A vesicle bioreactor as a step toward an artificial cell assembly. Proc. Natl. Acad. Sci. USA.

[5] Chen I.A., Salehi-Ashtiani K., Szostak J.W. (2005). RNA catalysis in model protocell vesicles. J. Am. Chem. Soc..

[6] Luisi P.L., Ferri F., Stano P. (2006). Approaches to semi-synthetic minimal cells: A review. Naturwissenschaften.

[7] Mansy S.S., Szostak J.W. (2009). Reconstructing the emergence of cellular life through the synthesis of model protocells. Cold Spring Harb. Symp. Quant. Biol..

[8] Ichihashi N., Matsuura T., Kita H., Sunami T., Suzuki H., Yomo T. (2010). Constructing partial models of cells. Cold Spring Harb. Perspect. Biol..

[9] Stano P., Carrara P., Kuruma Y., de Souza T.P., Luisi P.L. (2011). Compartmentalized reactions as a case of soft-matter biotechnology: Synthesis of proteins and nucleic acids inside lipid vesicles. J. Mater. Chem..

[10] Dzieciol A.J., Mann S. (2012). Designs for life: Protocell models in the laboratory. Chem. Soc. Rev..

[11] Torino D., Martini L., Mansy S.S. (2013). Piecing Together Cell-like Systems. Curr. Org. Chem..

[12] Nourian Z., Scott A., Danelon C. (2014). Toward the assembly of a minimal divisome. Syst. Synth. Biol..

[13] Blain J.C., Szostak J.W. (2014). Progress Toward Synthetic Cells. Ann. Rev. Biochem..

[14] Kurihara K., Okura Y., Matsuo M., Toyota T., Suzuki K., Sugawara T. (2015). A recursive vesicle-based model protocell with a primitive model cell cycle. Nat. Commun..

[15] Ichihashi N., Yomo T. (2016). Constructive Approaches for Understanding the Origin of Self-Replication and Evolution. Life.

[16] Salehi-Reyhani A., Ces O., Elani Y. (2017). Artificial cell mimics as simplified models for the study of cell biology. Exp. Biol. Med. (Maywood).

[17] Adamala K.P., Martin-Alarcon D.A., Guthrie-Honea K.R., Boyden E.S. (2017). Engineering genetic circuit interactions within and between synthetic minimal cells. Nat. Chem..

[18] Schwille P., Spatz J., Landfester K., Bodenschatz E., Herminghaus S., Sourjik V., Erb T.J., Bastiaens P., Lipowsky R., Hyman A. (2018). MaxSynBio: Avenues Towards Creating Cells from the Bottom Up. Angew. Chem. Int. Ed. Engl..

[19] Göpfrich K., Platzman I., Spatz J.P. (2018). Mastering Complexity: Towards Bottom-up Construction of Multifunctional Eukaryotic Synthetic Cells. Trends Biotechnol..

[20] Spoelstra W.K., Deshpande S., Dekker C. (2018). Tailoring the appearance: What will synthetic cells look like?. Curr. Opin. Biotechnol..

[21] Chandrawati R., Caruso F. (2012). Biomimetic liposome- and polymersome-based multicompartmentalized assemblies. Langmuir.

[22] Brea R.J., Hardy M.D., Devaraj N.K. (2015). Towards self-assembled hybrid artificial cells: Novel bottom-up approaches to functional synthetic membranes. Chemistry.

[23] Rideau E., Dimova R., Schwille P., Wurm F.R., Landfester K. (2018). Liposomes and polymersomes: A comparative review towards cell mimicking. Chem. Soc. Rev..

[24] Szostak J.W., Bartel D.P., Luisi P.L. (2001). Synthesizing life. Nature.

[25] Deplazes A., Huppenbauer M. (2009). Synthetic organisms and living machines: Positioning the products of synthetic biology at the borderline between living and non-living matter. Syst. Synth. Biol..

[26] Luisi P.L., Varela F.J. (1989). Self-replicating micelles—A chemical version of a minimal autopoietic system. Orig. Life Evol. Biosph..

[27] Bachmann P., Walde P., Luisi P., Lang J. (1990). Self-replicating reverse micelles and chemical autopoiesis. J. Am. Chem. Soc..

[28] Schmidli P.K., Schurtenberger P., Luisi P.L. (1991). Liposome-mediated enzymatic synthesis of phosphatidylcholine as an approach to self-replicating liposomes. J. Am. Chem. Soc..

[29] Walde P., Goto A., Monnard P., Wessicken M., Luisi P. (1994). Oparins Reactions Revisited—Enzymatic-Synthesis of Poly(adenylic Acid). J. Am. Chem. Soc..

[30] Oberholzer T., Wick R., Luisi P.L., Biebricher C.K. (1995). Enzymatic RNA replication in self-reproducing vesicles: An approach to a minimal cell. Biochem. Biophys. Res. Commun..

[31] Oberholzer T., Albrizio M., Luisi P. (1995). Polymerase Chain-Reaction in Liposomes. Chem. Biol..

[32] Oberholzer T., Nierhaus K.H., Luisi P.L. (1999). Protein expression in liposomes. Biochem. Biophys. Res. Commun..

[33] Oparin A.I. (1965). The pathways of the primary development of metabolism and artificial modeling of this development in coacervate drops. The Origins of Prebiological Systems and of Their Molecular Matrices.

[34] Chakrabarti A.C., Breaker R.R., Joyce G.F., Deamer D.W. (1994). Production of RNA by a polymerase protein encapsulated within phospholipid vesicles. J. Mol. Evol..

[35] Li M., Huang X., Tang T.-Y.D., Mann S. (2014). Synthetic cellularity based on non-lipid micro-compartments and protocell models. Curr. Opin. Chem. Biol..

[36] Dora Tang T.-Y., van Swaay D., deMello A., Ross Anderson J.L., Mann S. (2015). In vitro gene expression within membrane-free coacervate protocells. Chem. Commun. (Camb.).

[37] Frankel E.A., Bevilacqua P.C., Keating C.D. (2016). Polyamine/Nucleotide Coacervates Provide Strong Compartmentalization of Mg^2+^, Nucleotides, and RNA. Langmuir.

[38] Budin I., Szostak J.W. (2011). Physical effects underlying the transition from primitive to modern cell membranes. Proc. Natl. Acad. Sci. USA.

[39] Stano P. (2011). Minimal cells: Relevance and interplay of physical and biochemical factors. Biotechol. J..

[40] Engelhart A.E., Adamala K.P., Szostak J.W. (2016). A simple physical mechanism enables homeostasis in primitive cells. Nat. Chem..

[41] Luisi P.L. (2006). The Emergence of Life: From Chemical Origins to Synthetic Biology.

[42] Forster A.C., Church G.M. (2006). Towards synthesis of a minimal cell. Mol. Syst. Biol..

[43] Villarreal F., Tan C. (2017). Cell-free systems in the new age of synthetic biology. Front. Chem. Sci. Eng..

[44] Garenne D., Noireaux V. (2018). Cell-free transcription-translation: Engineering biology from the nanometer to the millimeter scale. Curr. Opin. Biotechnol..

[45] Forster A.C., Church G.M. (2007). Synthetic biology projects in vitro. Genome Res..

[46] Shi T., Han P., You C., Zhang Y.-H.P.J. (2018). An in vitro synthetic biology platform for emerging industrial biomanufacturing: Bottom-up pathway design. Synth. Syst. Biotechnol..

[47] Luisi P.L. (2007). Chemical Aspects of Synthetic Biology. Chem. Biodiv..

[48] Ashkenasy G., Hermans T.M., Otto S., Taylor A.F. (2017). Systems chemistry. Chem. Soc. Rev..

[49] Altamura E., Carrara P., D’Angelo F., Mavelli F., Stano P. (2018). Extrinsic stochastic factors (solute partition) in gene expression inside lipid vesicles and lipid-stabilized water-in-oil droplets: A review. Synth. Biol..

[50] Yu W., Sato K., Wakabayashi M., Nakaishi T., Ko-Mitamura E.P., Shima Y., Urabe I., Yomo T. (2001). Synthesis of functional protein in liposome. J. Biosci. Bioeng..

[51] Oberholzer T., Luisi P.L. (2002). The use of liposomes for constructing cell models. J. Biol. Phys..

[52] Ishikawa K., Sato K., Shima Y., Urabe I., Yomo T. (2004). Expression of a cascading genetic network within liposomes. FEBS Lett..

[53] Szoka F., Papahadjopoulos D. (1980). Comparative properties and methods of preparation of lipid vesicles (liposomes). Annu. Rev. Biophys. Bioeng..

[54] New R.R.C. (1990). Liposomes: A Practical Approach.

[55] Walde P., Nalwa H.S. (2004). Preparation of Vesicles (Liposomes). Encyclopedia of Nanoscience and Nanotechnology.

[56] Walde P., Cosentino K., Engel H., Stano P. (2010). Giant vesicles: Preparations and applications. ChemBioChem.

[57] Sato K., Obinata K., Sugawara T., Urabe I., Yomo T. (2006). Quantification of structural properties of cell-sized individual liposomes by flow cytometry. J. Biosci. Bioeng..

[58] Nishimura K., Hosoi T., Sunami T., Toyota T., Fujinami M., Oguma K., Matsuura T., Suzuki H., Yomo T. (2009). Population analysis of structural properties of giant liposomes by flow cytometry. Langmuir.

[59] Sakakura T., Nishimura K., Suzuki H., Yomo T. (2012). Statistical analysis of discrete encapsulation of nanomaterials in colloidal capsules. Anal. Methods.

[60] Luisi P.L., Walde P. (2000). Giant Vesicles.

[61] Fenz S.F., Sengupta K. (2012). Giant vesicles as cell models. Integr. Biol. (Camb.).

[62] Xiao Z., Huang N., Xu M., Lu Z., Wei Y. (1998). Novel Preparation of Asymmetric Liposomes with Inner and Outer Layer of Different Materials. Chem. Lett..

[63] Pautot S., Frisken B.J., Weitz D.A. (2003). Production of unilamellar vesicles using an inverted emulsion. Langmuir.

[64] Pautot S., Frisken B.J., Weitz D.A. (2003). Engineering asymmetric vesicles. Proc. Natl. Acad. Sci. USA.

[65] Dimova R., Marques C. (2019). The Giant Vesicle Book.

[66] Fujii S., Matsuura T., Sunami T., Nishikawa T., Kazuta Y., Yomo T. (2014). Liposome display for in vitro selection and evolution of membrane proteins. Nat. Protoc..

[67] Rampioni G., D’Angelo F., Messina M., Zennaro A., Kuruma Y., Tofani D., Leoni L., Stano P. (2018). Synthetic cells produce a quorum sensing chemical signal perceived by Pseudomonas aeruginosa. Chem. Commun..

[68] Fayolle D., Altamura E., D’Onofrio A., Madanamothoo W., Fenet B., Mavelli F., Buchet R., Stano P., Fiore M., Strazewski P. (2017). Crude phosphorylation mixtures containing racemic lipid amphiphiles self-assemble to give stable primitive compartments. Sci. Rep..

[69] Deshpande S., Caspi Y., Meijering A.E.C., Dekker C. (2016). Octanol-assisted liposome assembly on chip. Nat. Commun..

[70] Hong S.H., Kwon Y.-C., Jewett M.C. (2014). Non-standard amino acid incorporation into proteins using *Escherichia coli* cell-free protein synthesis. Front. Chem..

[71] Shimizu Y., Inoue A., Tomari Y., Suzuki T., Yokogawa T., Nishikawa K., Ueda T. (2001). Cell-free translation reconstituted with purified components. Nat. Biotechnol..

[72] Shimizu Y., Kanamori T., Ueda T. (2005). Protein synthesis by pure translation systems. Methods.

[73] Damiano L., Stano P. (2018). Synthetic Biology and Artificial Intelligence. Grounding a cross-disciplinary approach to the synthetic exploration of (embodied) cognition. Complex Syst..

[74] Hillebrecht J.R., Chong S. (2008). A comparative study of protein synthesis in in vitro systems: From the prokaryotic reconstituted to the eukaryotic extract-based. BMC Biotechnol..

[75] Koonin E.V. (2000). How many genes can make a cell: The minimal-gene-set concept. Annu. Rev. Genom. Hum. Genet..

[76] Gil R., Silva F.J., Peretó J., Moya A. (2004). Determination of the core of a minimal bacterial gene set. Microbiol. Mol. Biol. Rev..

[77] Caschera F., Noireaux V. (2014). Synthesis of 2.3 mg/mL of protein with an all *Escherichia coli* cell-free transcription-translation system. Biochimie.

[78] Villarreal F., Contreras-Llano L.E., Chavez M., Ding Y., Fan J., Pan T., Tan C. (2018). Synthetic microbial consortia enable rapid assembly of pure translation machinery. Nat. Chem. Biol..

[79] Van Swaay D., deMello A. (2013). Microfluidic methods for forming liposomes. Lab Chip.

[80] Trantidou T., Friddin M.S., Salehi-Reyhani A., Ces O., Elani Y. (2018). Droplet microfluidics for the construction of compartmentalised model membranes. Lab Chip.

[81] Deshpande S., Dekker C. (2018). On-chip microfluidic production of cell-sized liposomes. Nat. Protoc..

[82] Booth M.J., Schild V.R., Graham A.D., Olof S.N., Bayley H. (2016). Light-activated communication in synthetic tissues. Sci. Adv..

[83] Martino C., Kim S.-H., Horsfall L., Abbaspourrad A., Rosser S.J., Cooper J., Weitz D.A. (2012). Protein Expression, Aggregation, and Triggered Release from Polymersomes as Artificial Cell-like Structures. Angew. Chem. Int. Ed..

[84] Petit J., Thomi L., Schultze J., Makowski M., Negwer I., Koynov K., Herminghaus S., Wurm F.R., Bäumchen O., Landfester K. (2018). A modular approach for multifunctional polymersomes with controlled adhesive properties. Soft Matter.

[85] Jahn A., Vreeland W.N., Gaitan M., Locascio L.E. (2004). Controlled vesicle self-assembly in microfluidic channels with hydrodynamic focusing. J. Am. Chem. Soc..

[86] Siegal-Gaskins D., Tuza Z.A., Kim J., Noireaux V., Murray R.M. (2014). Gene circuit performance characterization and resource usage in a cell-free “breadboard”. ACS Synth. Biol..

[87] Karzbrun E., Shin J., Bar-Ziv R.H., Noireaux V. (2011). Coarse-grained dynamics of protein synthesis in a cell-free system. Phys. Rev. Lett..

[88] Stögbauer T., Windhager L., Zimmer R., Rädler J.O. (2012). Experiment and mathematical modeling of gene expression dynamics in a cell-free system. Integr. Biol..

[89] Calviello L., Stano P., Mavelli F., Luisi P.L., Marangoni R. (2013). Quasi-cellular systems: Stochastic simulation analysis at nanoscale range. BMC Bioinf..

[90] Mavelli F., Marangoni R., Stano P. (2015). A Simple Protein Synthesis Model for the PURE System Operation. Bull. Math. Biol..

[91] Matsuura T., Tanimura N., Hosoda K., Yomo T., Shimizu Y. (2017). Reaction dynamics analysis of a reconstituted *Escherichia coli* protein translation system by computational modeling. Proc. Natl. Acad. Sci. USA.

[92] Matsuura T., Hosoda K., Shimizu Y. (2018). Robustness of a Reconstituted *Escherichia coli* Protein Translation System Analyzed by Computational Modeling. ACS Synth. Biol..

[93] Mavelli F. (2012). Stochastic simulations of minimal cells: The Ribocell model. BMC Bioinf..

[94] Lazzerini-Ospri L., Stano P., Luisi P., Marangoni R. (2012). Characterization of the emergent properties of a synthetic quasi-cellular system. BMC Bioinf..

[95] Kapsner K., Simmel F.C. (2015). Partitioning Variability of a Compartmentalized In Vitro Transcriptional Thresholding Circuit. ACS Synth. Biol..

[96] Fanti A., Gammuto L., Mavelli F., Stano P., Marangoni R. (2018). Do protocells preferentially retain macromolecular solutes upon division/fragmentation? A study based on the extrusion of POPC giant vesicles. Integr. Biol. (Camb.).

[97] Rampioni G., Mavelli F., Damiano L., D’Angelo F., Messina M., Leoni L., Stano P. (2014). A synthetic biology approach to bio-chem-ICT: First moves towards chemical communication between synthetic and natural cells. Nat. Comput..

[98] Bozic B., Svetina S. (2004). A relationship between membrane properties forms the basis of a selectivity mechanism for vesicle self-reproduction. Eur. Biophys. J..

[99] Mavelli F., Ruiz-Mirazo K. (2010). ENVIRONMENT: A computational platform to stochastically simulate reacting and self-reproducing lipid compartments. Phys. Biol..

[100] Pereira de Souza T., Stano P., Luisi P.L. (2009). The minimal size of liposome-based model cells brings about a remarkably enhanced entrapment and protein synthesis. ChemBioChem.

[101] Luisi P.L., Allegretti M., Pereira de Souza T., Steiniger F., Fahr A., Stano P. (2010). Spontaneous protein crowding in liposomes: A new vista for the origin of cellular metabolism. ChemBioChem.

[102] Van Hoof B., Markvoort A.J., van Santen R.A., Hilbers P.A.J. (2012). On protein crowding and bilayer bulging in spontaneous vesicle formation. J. Phys. Chem. B.

[103] Paradisi P., Allegrini P., Chiarugi D. (2015). A renewal model for the emergence of anomalous solute crowding in liposomes. BMC Syst. Biol..

[104] Liu Y., Tsao C.-Y., Kim E., Tschirhart T., Terrell J.L., Bentley W.E., Payne G.F. (2017). Using a Redox Modality to Connect Synthetic Biology to Electronics: Hydrogel-Based Chemo-Electro Signal Transduction for Molecular Communication. Adv. Healthc. Mater..

[105] Selberg J., Gomez M., Rolandi M. (2018). The Potential for Convergence between Synthetic Biology and Bioelectronics. Cell. Syst..

[106] Bachmann P., Luisi P., Lang J. (1992). Autocatalytic Self-Replicating Micelles as Models for Prebiotic Structures. Nature.

[107] Walde P., Wick R., Fresta M., Mangone A., Luisi P. (1994). Autopoietic Self-Reproduction of Fatty-Acid Vesicles. J. Am. Chem. Soc..

[108] Varela F.G., Maturana H.R., Uribe R. (1974). Autopoiesis: The organization of living systems, its characterization and a model. Biosystems.

[109] Luisi P.L. (2003). Autopoiesis: A review and a reappraisal. Naturwissenschaften.

[110] Stano P. (2010). Synthetic biology of minimal living cells: Primitive cell models and semi-synthetic cells. Syst. Synth. Biol..

[111] Stano P., Luisi P.L. (2010). Achievements and open questions in the self-reproduction of vesicles and synthetic minimal cells. Chem. Commun. (Camb.).

[112] Letelier J.C., Marín G., Mpodozis J. (2003). Autopoietic and (M,R) systems. J. Theor. Biol..

[113] McMullin B. (2004). Thirty years of computational autopoiesis: A review. Artif. Life.

[114] Wikimedia Commons “Advanced Automation for Space Missions Figure 5-29.gif”. https://commons.wikimedia.org/w/index.php?curid=1687447.

[115] Gánti T. (1975). Organization of chemical reactions into dividing and metabolizing units: The chemotons. Biosystems.

[116] Gànti T. (2003). Chemoton Theory: Theory of Living Systems.

[117] (2013). Studies in History and Philosophy of Science Part C: Studies in History and Philosophy of Biological and Biomedical Sciences.

[118] Damiano L., Stano P. (2017). Understanding Embodied Cognition by Building Models of Minimal Life. Artificial Life and Evolutionary Computation.

[119] Bitbol M., Luisi P.L. (2004). Autopoiesis with or without cognition: Defining life at its edge. J. R. Soc. Interface.

[120] Bourgine P., Stewart J. (2004). Autopoiesis and cognition. Artif. Life.

[121] Ceruti M., Damiano L. (2018). Plural Embodiment(s) of Mind. Genealogy and Guidelines for a Radically Embodied Approach to Mind and Consciousness. Front. Psychol..

[122] Stano P., Rampioni G., D’Angelo F., Altamura E., Mavelli F., Marangoni R., Rossi F., Damiano L. (2018). Current Directions in Synthetic Cell Research. Advances in Bionanomaterials.

[123] Rigaud J.-L., Lévy D. (2003). Reconstitution of membrane proteins into liposomes. Meth. Enzymol..

[124] Dezi M., Cicco A.D., Bassereau P., Lévy D. (2013). Detergent-mediated incorporation of transmembrane proteins in giant unilamellar vesicles with controlled physiological contents. Proc. Natl. Acad. Sci. USA.

[125] Jørgensen I.L., Kemmer G.C., Pomorski T.G. (2017). Membrane protein reconstitution into giant unilamellar vesicles: A review on current techniques. Eur. Biophys. J..

[126] Robertson J.L. (2018). The lipid bilayer membrane and its protein constituents. J. Gen. Physiol..

[127] Miller C., Racker E., O’Brien R.D. (1979). Reconstitution of Membrane Transport Functions. The Receptors. General Principles and Procedures (A Comprehensive Treatise).

[128] Kuruma Y., Stano P., Ueda T., Luisi P.L. (2009). A synthetic biology approach to the construction of membrane proteins in semi-synthetic minimal cells. Biochim. Biophys. Acta.

[129] Hamada S., Tabuchi M., Toyota T., Sakurai T., Hosoi T., Nomoto T., Nakatani K., Fujinami M., Kanzaki R. (2014). Giant vesicles functionally expressing membrane receptors for an insect pheromone. Chem. Commun. (Camb.).

[130] Soga H., Fujii S., Yomo T., Kato Y., Watanabe H., Matsuura T. (2014). In vitro membrane protein synthesis inside cell-sized vesicles reveals the dependence of membrane protein integration on vesicle volume. ACS Synth. Biol..

[131] Ohta N., Kato Y., Watanabe H., Mori H., Matsuura T. (2016). In vitro membrane protein synthesis inside Sec translocon-reconstituted cell-sized liposomes. Sci. Rep..

[132] Uyeda A., Nakayama S., Kato Y., Watanabe H., Matsuura T. (2016). Construction of an in Vitro Gene Screening System of the *E. coli* EmrE Transporter Using Liposome Display. Anal. Chem..

[133] Furusato T., Horie F., Matsubayashi H.T., Amikura K., Kuruma Y., Ueda T. (2018). De Novo Synthesis of Basal Bacterial Cell Division Proteins FtsZ, FtsA, and ZipA Inside Giant Vesicles. ACS Synth. Biol..

[134] Yanagisawa M., Iwamoto M., Kato A., Yoshikawa K., Oiki S. (2011). Oriented Reconstitution of a Membrane Protein in a Giant Unilamellar Vesicle: Experimental Verification with the Potassium Channel KcsA. J. Am. Chem. Soc..

[135] Altamura E., Milano F., Tangorra R.R., Trotta M., Omar O.H., Stano P., Mavelli F. (2017). Highly oriented photosynthetic reaction centers generate a proton gradient in synthetic protocells. Proc. Natl. Acad. Sci. USA.

[136] Sachse R., Dondapati S.K., Fenz S.F., Schmidt T., Kubick S. (2014). Membrane protein synthesis in cell-free systems: From bio-mimetic systems to bio-membranes. FEBS Lett..

[137] Kuruma Y., Suzuki T., Ono S., Yoshida M., Ueda T. (2012). Functional analysis of membranous Fo-a subunit of F1Fo-ATP synthase by in vitro protein synthesis. Biochem. J..

[138] Choi H.-J., Montemagno C.D. (2005). Artificial Organelle: ATP Synthesis from Cellular Mimetic Polymersomes. Nano Lett..

[139] Feng X., Jia Y., Cai P., Fei J., Li J. (2016). Coassembly of Photosystem II and ATPase as Artificial Chloroplast for Light-Driven ATP Synthesis. ACS Nano.

[140] Altamura E., Fiorentino R., Milano F., Trotta M., Palazzo G., Stano P., Mavelli F. (2017). First moves towards photoautotrophic synthetic cells: In Vitro study of photosynthetic reaction centre and cytochrome bc1 complex interactions. Biophys. Chem..

[141] Kumar B.V.V.S.P., Fothergill J., Bretherton J., Tian L., Patil A.J., Davis S.A., Mann S. (2018). Chloroplast-containing coacervate micro-droplets as a step towards photosynthetically active membrane-free protocells. Chem. Commun..

[142] Lee K.Y., Park S.-J., Lee K.A., Kim S.-H., Kim H., Meroz Y., Mahadevan L., Jung K.-H., Ahn T.K., Parker K.K. (2018). Photosynthetic artificial organelles sustain and control ATP-dependent reactions in a protocellular system. Nat. Biotechnol..

[143] Kim T., Murdande S., Gruber A., Kim S. (1996). Sustained-release Morphine for Epidural Analgesia in Rats. Anesthesiology.

[144] Kisak E.T., Coldren B., Zasadzinski J.A. (2002). Nanocompartments Enclosing Vesicles, Colloids, and Macromolecules via Interdigitated Lipid Bilayers. Langmuir.

[145] Kisak E.T., Coldren B., Evans C.A., Boyer C., Zasadzinski J.A. (2004). The vesosome—A multicompartment drug delivery vehicle. Curr. Med. Chem..

[146] Walker S.A., Kennedy M.T., Zasadzinski J.A. (1997). Encapsulation of bilayer vesicles by self-assembly. Nature.

[147] Bolinger P.-Y., Stamou D., Vogel H. (2008). An integrated self-assembled nanofluidic system for controlled biological chemistries. Angew. Chem. Int. Ed. Engl..

[148] Paleos C.M., Tsiourvas D., Sideratou Z. (2011). Interaction of vesicles: Adhesion, fusion and multicompartment systems formation. ChemBioChem.

[149] Hadorn M., Boenzli E., Eggenberger Hotz P., Hanczyc M.M. (2012). Hierarchical Unilamellar Vesicles of Controlled Compositional Heterogeneity. PLoS ONE.

[150] Deng N.-N., Yelleswarapu M., Zheng L., Huck W.T.S. (2017). Microfluidic Assembly of Monodisperse Vesosomes as Artificial Cell Models. J. Am. Chem. Soc..

[151] Haller B., Göpfrich K., Schröter M., Janiesch J.-W., Platzman I., Spatz J.P. (2018). Charge-controlled microfluidic formation of lipid-based single- and multicompartment systems. Lab Chip.

[152] Pak C.C., Ali S., Janoff A.S., Meers P. (1998). Triggerable liposomal fusion by enzyme cleavage of a novel peptide–lipid conjugate. Biochim. Biophys. Acta (BBA) Biomembr..

[153] Chaize B., Nguyen M., Ruysschaert T., le Berre V., Trévisiol E., Caminade A.-M., Majoral J.P., Pratviel G., Meunier B., Winterhalter M. (2006). Microstructured liposome array. Bioconjug. Chem..

[154] Liu X., Zhao R., Zhang Y., Jiang X., Yue J., Jiang P., Zhang Z. (2007). Using giant unilamellar lipid vesicle micro-patterns as ultrasmall reaction containers to observe reversible ATP synthesis/hydrolysis of F0F1-ATPase directly. Biochim. Biophys. Acta.

[155] Christensen S.M., Stamou D.G. (2010). Sensing-applications of surface-based single vesicle arrays. Sensors.

[156] Osaki T., Kamiya K., Kawano R., Sasaki H., Takeuchi S. Towards artificial cell array system: Encapsulation and hydration technologies integrated in liposome array. Proceedings of the 2012 IEEE 25th International Conference on Micro Electro Mechanical Systems (MEMS).

[157] Kang Y.J., Wostein H.S., Majd S. (2013). A simple and versatile method for the formation of arrays of giant vesicles with controlled size and composition. Adv. Mater..

[158] Mantri S., Sapra K.T. (2013). Evolving protocells to prototissues: Rational design of a missing link. Biochem. Soc. Trans..

[159] Hamano H., Tonooka T., Osaki T., Takeuchi S. Highly packed liposome assemblies toward synthetic tissue. Proceedings of the 2014 IEEE 27th International Conference on Micro Electro Mechanical Systems (MEMS).

[160] Hamano H., Osaki T., Takeuchi S. Liposome arrangement connected with avidin-biotin complex for constructing functional synthetic tissue. Proceedings of the 2015 28th IEEE International Conference on Micro Electro Mechanical Systems (MEMS).

[161] Kazayama Y., Teshima T., Osaki T., Takeuchi S., Toyota T. (2016). Integrated Microfluidic System for Size-Based Selection and Trapping of Giant Vesicles. Anal. Chem..

[162] Hadorn M., Hotz P.E. (2010). DNA-Mediated Self-Assembly of Artificial Vesicles. PLoS ONE.

[163] Hadorn M., Boenzli E., Sørensen K.T., De Lucrezia D., Hanczyc M.M., Yomo T. (2013). Defined DNA-Mediated Assemblies of Gene-Expressing Giant Unilamellar Vesicles. Langmuir.

[164] Carrara P., Stano P., Luisi P.L. (2012). Giant Vesicles “Colonies”: A Model for Primitive Cell Communities. ChemBioChem.

[165] Nakano T., Eckford A.W., Haraguchi T. (2013). Molecular Communications.

[166] Nakano T. (2017). Molecular Communication: A 10 Year Retrospective. IEEE Trans. Mol. Biol. Multi-Scale Commun..

[167] Amos M., Dittrich P., McCaskill J., Rasmussen S. (2011). Biological and Chemical Information Technologies. Procedia Comput. Sci..

[168] Stano P., Rampioni G., Carrara P., Damiano L., Leoni L., Luisi P.L. (2012). Semi-synthetic minimal cells as a tool for biochemical ICT. BioSystems.

[169] Cronin L., Krasnogor N., Davis B.G., Alexander C., Robertson N., Steinke J.H.G., Schroeder S.L.M., Khlobystov A.N., Cooper G., Gardner P.M. (2006). The imitation game—A computational chemical approach to recognizing life. Nat. Biotechnol..

[170] Leduc P.R., Wong M.S., Ferreira P.M., Groff R.E., Haslinger K., Koonce M.P., Lee W.Y., Love J.C., McCammon J.A., Monteiro-Riviere N.A. (2007). Towards an in vivo biologically inspired nanofactory. Nat. Nanotechnol..

[171] Gardner P.M., Winzer K., Davis B.G. (2009). Sugar synthesis in a protocellular model leads to a cell signalling response in bacteria. Nat. Chem..

[172] Kaneda M., Nomura S.M., Ichinose S., Kondo S., Nakahama K., Akiyoshi K., Morita I. (2009). Direct formation of proteo-liposomes by in vitro synthesis and cellular cytosolic delivery with connexin-expressing liposomes. Biomaterials.

[173] Rampioni G., D’Angelo F., Leoni L., Stano P. (2019). Gene-expressing liposomes as synthetic cells for molecular communication studies. Front. Bioeng. Biotech. Synth. Biol..

[174] Lentini R., Santero S.P., Chizzolini F., Cecchi D., Fontana J., Marchioretto M., Del Bianco C., Terrell J.L., Spencer A.C., Martini L. (2014). Integrating artificial with natural cells to translate chemical messages that direct *E. coli* behaviour. Nat. Commun..

[175] Lentini R., Martín N.Y., Forlin M., Belmonte L., Fontana J., Cornella M., Martini L., Tamburini S., Bentley W.E., Jousson O. (2017). Two-Way Chemical Communication between Artificial and Natural Cells. ACS Cent. Sci..

[176] Niederholtmeyer H., Chaggan C., Devaraj N.K. (2018). Communication and quorum sensing in non-living mimics of eukaryotic cells. Nat. Commun..

[177] Tang T.-Y.D., Cecchi D., Fracasso G., Accardi D., Coutable-Pennarun A., Mansy S.S., Perriman A.W., Anderson J.L.R., Mann S. (2018). Gene-Mediated Chemical Communication in Synthetic Protocell Communities. ACS Synth. Biol..

[178] Krinsky N., Kaduri M., Zinger A., Shainsky-Roitman J., Goldfeder M., Benhar I., Hershkovitz D., Schroeder A. (2018). Synthetic Cells Synthesize Therapeutic Proteins inside Tumors. Adv. Healthc. Mater..

[179] Ding Y., Contreras-Llano L.E., Morris E., Mao M., Tan C. (2018). Minimizing Context Dependency of Gene Networks Using Artificial Cells. ACS Appl. Mater. Interfaces.

[180] Lee S., Koo H., Na J.H., Lee K.E., Jeong S.Y., Choi K., Kim S.H., Kwon I.C., Kim K. (2014). DNA amplification in neutral liposomes for safe and efficient gene delivery. ACS Nano.

[181] Tsugane M., Suzuki H. (2018). Reverse Transcription Polymerase Chain Reaction in Giant Unilamellar Vesicles. Sci. Rep..

[182] Van Nies P., Westerlaken I., Blanken D., Salas M., Mencía M., Danelon C. (2018). Self-replication of DNA by its encoded proteins in liposome-based synthetic cells. Nat. Commun..

[183] Scott A., Noga M.J., de Graaf P., Westerlaken I., Yildirim E., Danelon C. (2016). Cell-Free Phospholipid Biosynthesis by Gene-Encoded Enzymes Reconstituted in Liposomes. PLoS ONE.

[184] Gell-Mann M., Lloyd S. (1996). Information measures, effective complexity, and total information. Complexity.

[185] Emmeche C. (1997). Aspects of Complexity in Life and Science. Philosophica.

[186] Lloyd S. (2001). Measures of complexity: A nonexhaustive list. IEEE Control Syst. Mag..

[187] Cheng Z.L., Luisi P.L. (2003). Coexistence and mutual competition of vesicles with different size distributions. J. Phys. Chem. B.

[188] Chen I.A., Roberts R.W., Szostak J.W. (2004). The emergence of competition between model protocells. Science.

[189] Adamala K., Szostak J.W. (2013). Competition between model protocells driven by an encapsulated catalyst. Nat. Chem..

[190] Stano P. (2007). Question 7: New aspects of interactions among vesicles. Orig. Life Evol. Biosph..

[191] Nishimura K., Tsuru S., Suzuki H., Yomo T. (2015). Stochasticity in Gene Expression in a Cell-Sized Compartment. ACS Synth. Biol..

[192] Stano P., de Souza T.P., Carrara P., Altamura E., D’Aguanno E., Caputo M., Luisi P.L., Mavelli F. (2015). Recent Biophysical Issues About the Preparation of Solute-Filled Lipid Vesicles. Mech. Adv. Mater. Struct..

[193] Damiano L., Hiolle A., Cañamero L., Lenaerts T., Giacobini M., Bersini H., Bourgine P., Dorigo M., Doursat R. (2011). Grounding Synthetic Knowledge. Advances in Artificial Life, ECAL 2011.

[194] Sunami T., Sato K., Matsuura T., Tsukada K., Urabe I., Yomo T. (2006). Femtoliter compartment in liposomes for in vitro selection of proteins. Anal. Biochem..

[195] Murtas G., Kuruma Y., Bianchini P., Diaspro A., Luisi P.L. (2007). Protein synthesis in liposomes with a minimal set of enzymes. Biochem. Biophys. Res. Commun..

[196] Dong H., Nilsson L., Kurland C.G. (1996). Co-variation of tRNA abundance and codon usage in *Escherichia coli* at different growth rates. J. Mol. Biol..

[197] Gibson D.G., Glass J.I., Lartigue C., Noskov V.N., Chuang R.-Y., Algire M.A., Benders G.A., Montague M.G., Ma L., Moodie M.M. (2010). Creation of a bacterial cell controlled by a chemically synthesized genome. Science.

[198] Hutchison C.A., Chuang R.-Y., Noskov V.N., Assad-Garcia N., Deerinck T.J., Ellisman M.H., Gill J., Kannan K., Karas B.J., Ma L. (2016). Design and synthesis of a minimal bacterial genome. Science.

[199] Jewett M.C., Fritz B.R., Timmerman L.E., Church G.M. (2013). In vitro integration of ribosomal RNA synthesis, ribosome assembly, and translation. Mol. Syst. Biol..

[200] Caschera F., Lee J.W., Ho K.K.Y., Liu A.P., Jewett M.C. (2016). Cell-free compartmentalized protein synthesis inside double emulsion templated liposomes with in vitro synthesized and assembled ribosomes. Chem. Commun. (Camb.).

[201] Li J., Haas W., Jackson K., Kuru E., Jewett M.C., Fan Z.H., Gygi S., Church G.M. (2017). Cogenerating Synthetic Parts toward a Self-Replicating System. ACS Synth. Biol..

[202] Liu Z., Zhang Y., Jia X., Hu M., Deng Z., Xu Y., Liu T. (2017). In Vitro Reconstitution and Optimization of the Entire Pathway to Convert Glucose into Fatty Acid. ACS Synth. Biol..

[203] Exterkate M., Caforio A., Stuart M.C.A., Driessen A.J.M. (2018). Growing Membranes In Vitro by Continuous Phospholipid Biosynthesis from Free Fatty Acids. ACS Synth. Biol..

[204] Blöchliger E., Blocher M., Walde P., Luisi P.L. (1998). Matrix effect in the size distribution of fatty acid vesicles. J. Phys. Chem. B.

[205] Lonchin S., Luisi P.L., Walde P., Robinson B.H. (1999). A matrix effect in mixed phospholipid/fatty acid vesicle formation. J. Phys. Chem. B.

[206] Berclaz N., Muller M., Walde P., Luisi P.L. (2001). Growth and transformation of vesicles studied by ferritin labeling and cryotransmission electron microscopy. J. Phys. Chem. B.

[207] Rasi S., Mavelli F., Luisi P.L. (2003). Cooperative micelle binding and matrix effect in oleate vesicle formation. J. Phys. Chem. B.

[208] Stano P., Wehrli E., Luisi P.L. (2006). Insights into the self-reproduction of oleate vesicles. J. Phys. Condens. Matter.

[209] Zhu T.F., Szostak J.W. (2009). Coupled Growth and Division of Model Protocell Membranes. J. Am. Chem. Soc..

[210] Carello C., Turvey M.T., Kugler P.N., Shaw R.E., Gazzaniga M.S. (1984). Inadequacies of the Computer Metaphor. Handbook of Cognitive Neuroscience.

[211] Danchin A. (2009). Bacteria as computers making computers. FEMS Microbiol. Rev..

[212] Shapiro E. (2012). A mechanical Turing machine: Blueprint for a biomolecular computer. Interface Focus.

[213] Nicholson D.J. (2013). Organisms≠Machines. Stud. Hist. Philos. Biol. Biomed. Sci..

[214] Boldt J. (2018). Machine metaphors and ethics in synthetic biology. Life Sci. Soc. Policy.

[215] Jiang Y., Xu C. (2010). The calculation of information and organismal complexity. Biol. Direct.

[216] Kolmogorov A.N. (1968). Three approaches to the quantitative definition of information. Int. J. Comp. Math..

[217] McCabe T.J. (1976). A complexity measure. IEEE Trans. Soft. Eng..

[218] Shin J., Noireaux V. (2012). An *E. coli* Cell-Free Expression Toolbox: Application to Synthetic Gene Circuits and Artificial Cells. ACS Synth. Biol..

